# Hydrogen Bonding and Infrared Spectra of Ethyl-3-methylimidazolium Bis(trifluoromethylsulfonyl)imide/Water Mixtures: A View from Molecular Dynamics Simulations

**DOI:** 10.1021/acs.jpcb.2c06947

**Published:** 2022-12-14

**Authors:** Piotr Wróbel, Piotr Kubisiak, Andrzej Eilmes

**Affiliations:** Faculty of Chemistry, Jagiellonian University, Gronostajowa 2, 30-387 Kraków, Poland

## Abstract

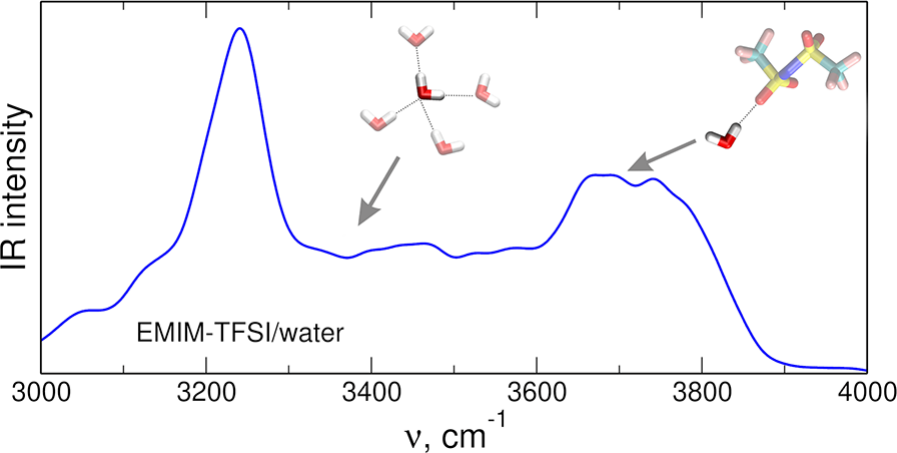

Simulations of ab initio molecular dynamics have been performed for mixtures of ethyl-3-methylimidazolium bis(trifluoromethylsulfonyl)imide (EMIM-TFSI) ionic liquid and water. Statistics of donors and acceptors of hydrogen bonds has revealed that with increasing water content, hydrogen bonds between EMIM cations and TFSI anions are replaced by bonds to water molecules. In the mixture of liquids, the total number of bonds (from EMIM cations or water molecules) formed by TFSI acceptors increases. IR spectra obtained from ab initio molecular dynamics trajectories are in good agreement with literature data for ionic liquid/water systems. Analysis of oscillations of individual C–H and O–H bonds has shown correlations between vibrational frequencies and hydrogen bonds formed by an EMIM cation or water molecule and has indicated that the changes in the IR spectrum result from the decreased number of water–water hydrogen bonds in the mixture. The tests of DFTB methodology with tailored parameterizations have yielded reasonably good description of the IR spectrum of bulk water, whereas available parameterizations have failed in satisfactory reproduction of the IR spectrum of EMIM-TFSI/water mixtures in the region above 3000 cm^–1^.

## 1 Introduction

Ionic liquids (ILs) attract significant attention owing to their physicochemical properties (which may be tailored by modifications of IL ions) and prospective applications as alternative solvents. ILs are organic salts molten at ambient temperatures; therefore, the interactions between ions of the IL are dominated by strong, long-range electrostatic interactions. Nevertheless, short-range specific interactions such as hydrogen bonds (HBs) can also occur in ILs, provided that appropriate hydrogen donors and acceptors are available in IL cations and anions. The importance of hydrogen bonding for the structure and solvation properties of ILs has been already documented.^1−5^

More possibilities of HB formation in ILs arise when a hydrogen bonding-capable molecular solvent is dissolved in the liquid. Some amounts of water are usually present in ILs in typical laboratory conditions due to the hydrophilic nature of many ILs. Therefore, numerous experimental or computational works focus on IL/water mixtures, their structure, and dynamics and on investigations on how the presence of water affects the structure and properties of the IL.^6−14^

The interactions in liquid phase and the hydrogen bonding are commonly studied via infrared (IR) or Raman spectroscopy. It is therefore unsurprising that vibrational spectroscopy has been applied to investigate water/IL systems and to detect changes in HB properties in such solutions.^10,15−25^ Water molecules have been suggested as vibrational probes into the structure and dynamics of ILs.^23^

Vibrational frequencies in IL/water solutions can be assessed from quantum-chemical calculations in a routine manner, from the analysis of the eigenvalues of the Hessian matrix at the optimized geometry of the system containing only few molecules/ions. Computations of such a type have been performed for clusters of IL ions and water molecules.^16,19,21,26−28^ On the other hand, vibrational spectra can be obtained from the ab initio molecular dynamics (AIMD) simulations, as Fourier transforms (FTs) of the autocorrelation function of the dipole moment (IR spectrum) or the polarizability (Raman spectrum) of the system. This methodology can be used not only in calculations for molecules or small aggregates but also for simulations of the spectrum of a bulk liquid. It has been applied for computations of the IR spectra of neat ILs^29−31^ and for mixtures of ILs with water.^32^

In this paper, we extended our AIMD study^33^ on an HB network in a typical aprotic IL, 1-ethyl-3-methylimidazolium bis(trifluoromethylsulfonyl)imide (EMIM-TFSI) to EMIM-TFSI/H_2_O mixtures. We analyzed the statistics of different HBs and how it is affected by the presence of water molecules. From AIMD trajectories, we obtained the IR spectra of systems with different water fractions and performed analysis of selected vibrations to check the correlations between local structure/formation of an HB and the observed vibrational frequencies. Finally, we tested the applicability of density functional-based tight binding (DFTB),^34,35^ less demanding computationally than the density functional theory (DFT)-based methodology used for the main AIMD simulations. The DFTB method has been already used for simulations of IL systems,^36−38^ including the analysis of HBs and IR spectra.^37^ Here, we assessed the performance of DFTB in the description of spectral effects related to hydrogen bonding in mixed IL/water systems.

## 2 Computational Details

The EMIM-TFSI/H_2_O systems simulated in this work consisted of 15 pairs of the IL ions and an increasing number of H_2_O molecules: 0, 2, 5, and 15, corresponding to water mole fraction *x* equal to 0, 0.12, 0.25, and 0.5, respectively. Additionally, simulations were performed for a neat water box containing 181 H_2_O molecules. Initial structures were prepared using Packmol software.^39^ Two independent replicas of the systems were simulated for neat liquids and three for IL/water mixtures in order to average results.

Initial MD simulations were performed in the NAMD v 2.12 simulation package.^40^ For the description of EMIM-TFSI, we used the nonpolarizable force field NP1 from our previous work^41^ with bonded parameters taken from Pádua et al.’s parametrization^42^ and nonbonded from Köddermann et al.’s force field.^43^ Atomic charges were not scaled. Water molecules were described using the 3-site flexible TIP3P water model based on the original work by Jorgensen et al.^44^

NAMD simulations were performed in the NpT and NVT ensembles at *p* = 1 atm and *T* = 298 K with Langevin dynamics and a modified Nose–Hoover Langevin barostat.^45,46^ A time step of 1 fs was used to integrate equations of motion. Periodic boundary conditions were applied to the system, and electrostatic interactions were taken into account via the particle mesh Ewald algorithm.^47^ First, 100 ns of equilibration were performed in NpT runs; then, we simulated another 100 ns of the trajectories in the NVT ensemble at the density obtained in the NpT part. The density obtained for the neat EMIM-TFSI was 1.518 g/cm^3^, almost exactly the experimental value 1.519 g/cm^3^.^48^ Densities of the systems with *x* = 0.12, 0.25, and 0.5 were 1.491, 1.485, and 1.462 g/cm^3^, respectively; the calculated densities decrease a little faster than measured values and therefore are 0.01–0.02 g/cm^3^ lower than the experimental data.^49^

Next, the structures from the classical MD simulations were used as starting points of AIMD in the CP2K package,^50,51^ employing the PBE functional with empirical dispersion correction D3,^52^ Goedecker’s pseudopotentials,^53^ and a molecularly optimized DZVP-MOLOPT-GTH basis set.^54^ AIMD simulations were performed for 40 ps in the NVT ensemble at *T* = 298 K with a time step of 1 fs using the Nosé–Hoover thermostat.

DFTB+ v. 22.1 software^55^ was used in the DFTB-based simulations. The application of the DFTB methodology is limited by the availability of the parameter sets. In this work, we used the publicly available parameterizations. For H_2_O molecules, these were *3ob*,^56,57^*matsci*,^58^*mio*,^34^ and *ob2*(59) general purpose sets. In addition, we tried also the *water-matsci* and *water-matsci-uff* parameterizations derived from the *matsci* set to improve the description of the structure and dynamics of bulk water.^60^ Recently, a general and broadly parameterized DFTB3 variant was presented, called GFN-xTB.^61,62^ In our simulations for neat water, we used two variants of this method, GFN1-xTB and GFN2-xTB. Only *3ob* and the general GFN-xTB sets of parameters cover all atoms of EMIM-TFSI ions. Therefore, simulations for neat IL and for *x* = 0.5 IL/water mixtures were performed only with *3ob* and GFN2-xTB sets; the GFN1-xTB was not used because for neat water, it produced results worse than GFN2-xTB (cf. Section 3.3). The NVT parameters and the length of DFTB+ trajectories were the same as for AIMD simulations in CP2K.

The last 30 ps of MD trajectories were used for analysis. Plots of distribution functions were prepared using TRAVIS.^63^ The IR spectra were obtained from AIMD and DFTB MD trajectories as the Fourier transforms of the dipole moment autocorrelation function. To produce smooth plots, the individual peaks were convoluted with Gaussian curves with σ = 15 cm^–1^. The distribution functions and calculated spectra were averaged over all replicas of the trajectories.

## 3 Results and Discussion

### 3.1 Structure of Liquids

**Figure 1:**
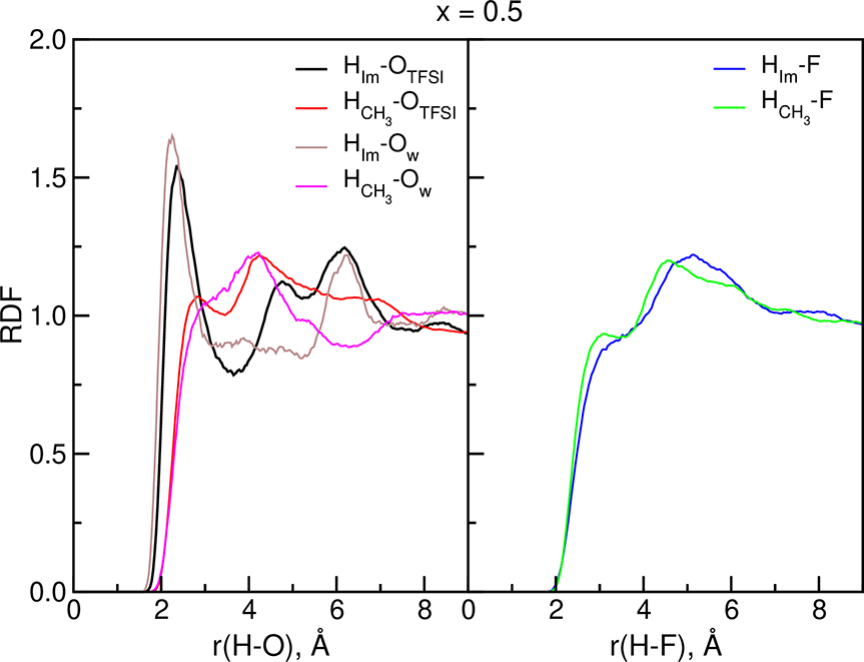
Radial distribution functions for selected atom pairs obtained from AIMD simulations for *x* = 0.5.

To gain some information on the structure of the liquid, we analyzed distribution functions between selected atoms. In the following, we will denote the oxygen atoms from TFSI anions as O_TFSI_ and those from water molecules as O_w_. Labeling of the atoms in the EMIM cation is shown in Scheme S1 in the Supporting Information. H_Im_ and H_CH3_ stand for H atoms bound to carbon atoms from the imidazolium ring or from the CH_3_ group, respectively. The carbon atoms from the imidazolium ring will be generally labeled C_Im_; the carbon atom located between two nitrogen atoms will be denoted as C_1_. Plots of radial distribution functions (RDFs) obtained from AIMD DFT simulations are shown in Figure 1 (for *x* = 0.5) and in Figure S1 in the Supporting Information (for *x* = 0). The first sharp maximum in the H_Im_–O_TFSI_ and in the H_Im_–O_w_ RDF appears slightly above 2 Å, followed by lower and broader maxima above 4 Å. On the other hand, the maxima between 2 and 3 Å in the H_CH3_–O_TFSI_ and H_CH3_–O_w_ RDFs are weak. The main maxima of these RDFs are located at the distance of about 4 Å and are lower than the first peak in the RDFs obtained for H_Im_ atoms. The picture is similar in the case of H_Im_–F and H_CH3_–F RDFs with weak features at 3 Å and the main maximum between 4.5 and 5.5 Å. From the RDFs, one may expect that EMIM cations will interact with oxygen atoms from anions or water molecules through the hydrogens of the imidazolium ring.

**Figure 2:**
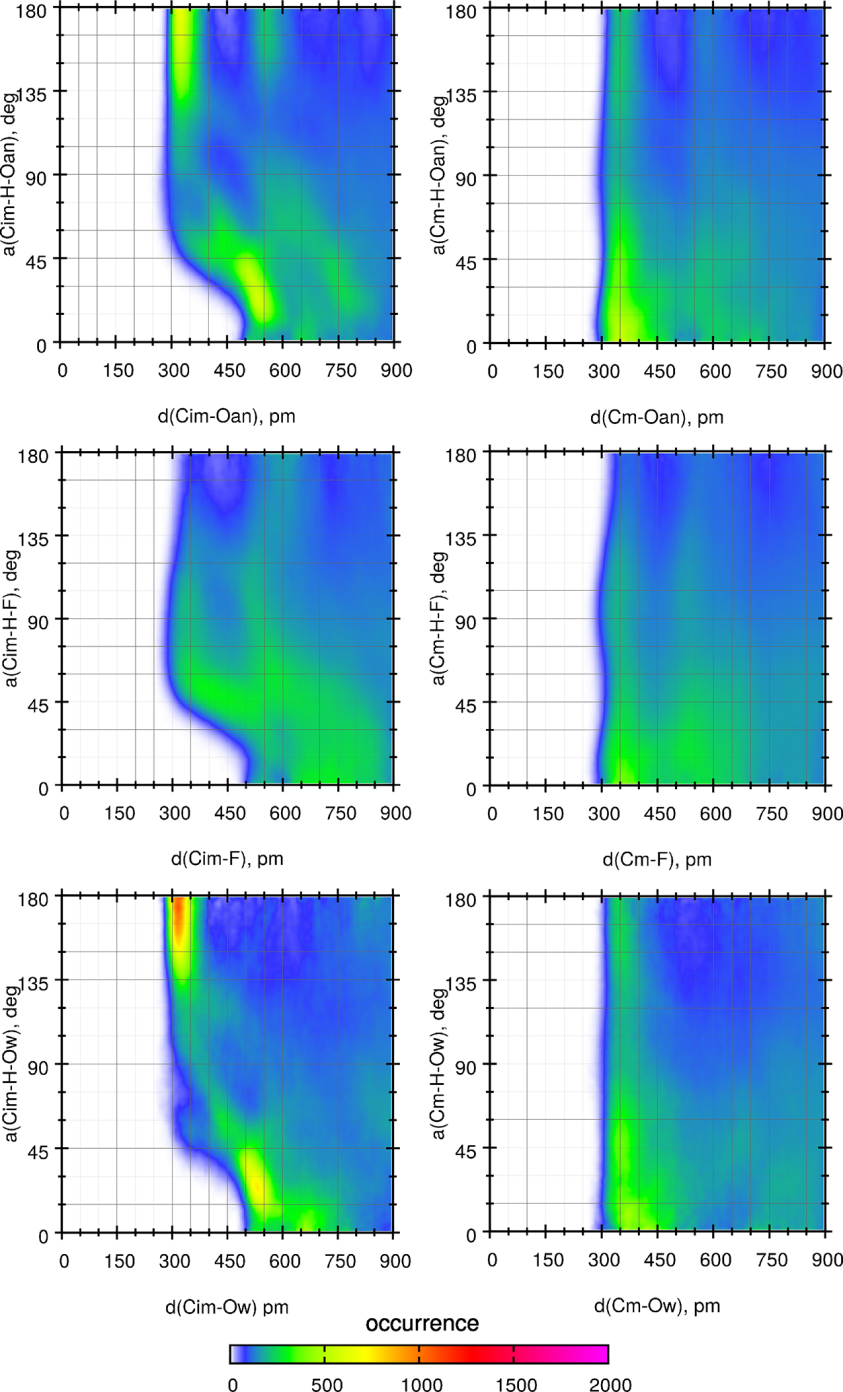
Combined distribution functions for selected D–H–A atoms in the *x* = 0.5 system. C_m_ denotes C atoms from CH_3_ groups, C_Im_ are the C atoms from the imidazolium ring, and O_an_ are the O atoms from TFSI anions.

Formation of a HB requires not only a small distance between hydrogen and the acceptor atom but also a sufficiently linear arrangement of the three atoms involved in the bond; the deviation from the linearity is the smallest for strong HBs and the largest for weak bonds.^2^ Therefore, we analyzed the combined distribution functions (CDFs) showing the relative probability of finding a configuration of atoms at specified D–A distance and D–H–A angle. Sample CDF plots for the *x* = 0.5 system are presented in Figure 2; the data for *x* = 0 and CDFs involving the C_1_ atom are shown in Figures S2 and S3 in the Supporting Information.

**Figure 3:**
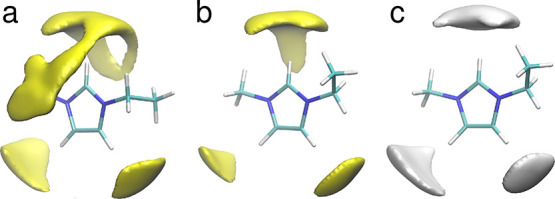
Spatial distribution functions of oxygen atoms around EMIM cations: TFSI ions in the neat IL (a); TFSI ions in the *x* = 0.5 mixture (b); water molecules in the *x* = 0.5 mixture (c). Surfaces of particle density 10 atoms/nm^3^ are shown.

The configurations of D–H–A atoms suitable for hydrogen bonding are these corresponding to the D–H–A angle close to 180° and to the D–A distances up to 350 pm. As seen in Figure 2, there is a region of increased probability of finding C_Im_–O_TFSI_ atom pair at the distance 300–350 pm and at the C_Im_–H–O_TFSI_ angle of 135–180°. The probability of finding such a configuration for C_m_–H–O_TFSI_ atoms is smaller but non-negligible. On the other hand, occurrences of C–H–F arrangements leading to HB are infrequent. We may also note that the C_Im_–H–O_w_ CDF has the maximum at about 300 pm and 180°, indicating the possibility of interactions of H_Im_ with water acceptors. Spatial distribution functions (SDFs) of TFSI and water oxygen atoms around EMIM cations, shown in Figure 3, confirm that there are regions of increased density of oxygen acceptors close to hydrogen atoms from the imidazolium ring; therefore, one may expect a significant number of HBs involving these D–A pairs.

In the analysis of hydrogen bonding in the MD trajectories, we used the following criteria of the existence of the D–H···A bond between the donor D and the acceptor A: (1) the D–A distance not exceeding 3.5 Å, and (2) the deviation of D–H–A atoms from the linearity by no more than 40°. The same thresholds were used in our previous work,^33^ where we showed that they lead to the H–A distances smaller than the sum of van der Waals radii of H and A atoms.

**Figure 4:**
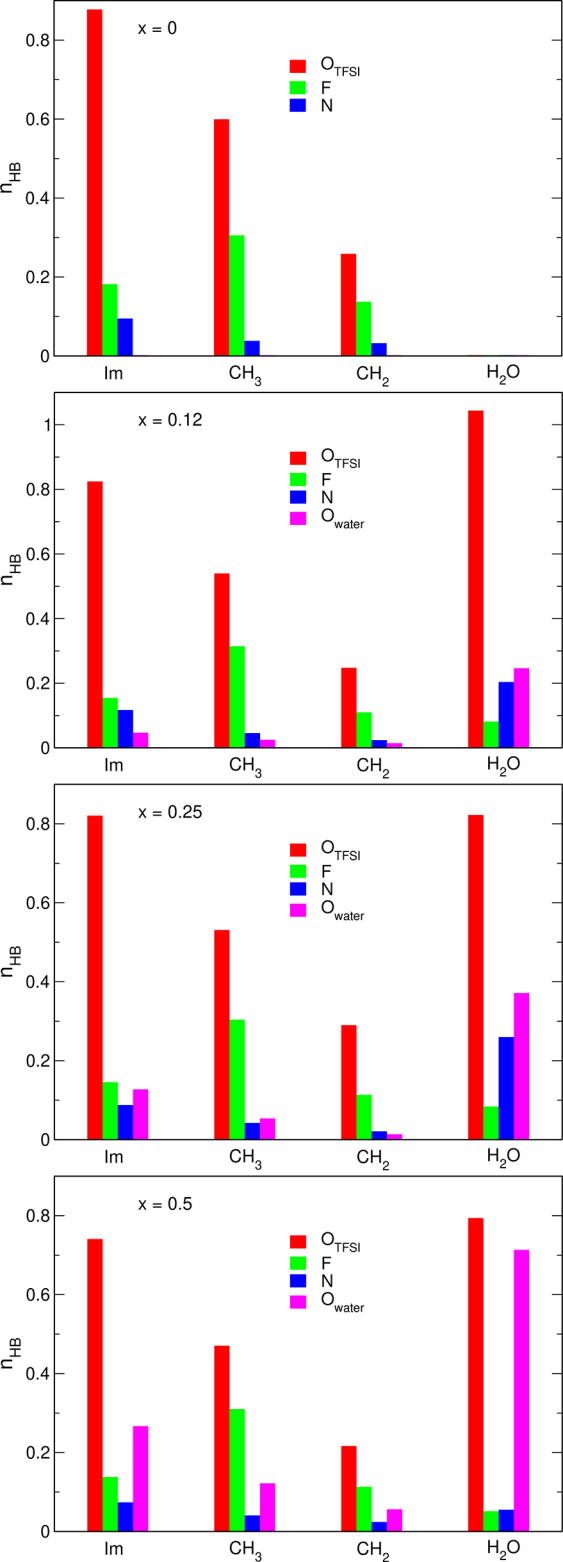
Statistics of hydrogen bonds obtained from AIMD simulations. Donors are shown in the horizontal axis; colors mark the acceptors.

In Figure 4, we summarize the breakdown of the average number of HBs according to different hydrogen donors (carbon atoms from the imidazolium ring, CH_2_ or CH_3_ group of the cation, and O atoms from water molecules) and acceptors (O_TFSI_, N, and F are the atoms from the anions, O_water_ are oxygen atoms from H_2_O molecules). The numbers were calculated per EMIM cation or per water molecule, depending on the donor. In the neat IL, EMIM cations form 2.5 HBs per cation (the sum of all contributions shown in the top panel of Figure 4), most of them to oxygen atoms (1.7 bonds per EMIM; the sum of red bars in Figure 4). About half of these H···O_TFSI_ bonds engages the imidazolium ring hydrogens. The average number of H_CH3_···O_TFSI_ bonds reaches 0.6 per cation; the lower probability of finding individual pair of H_CH3_ and O_TFSI_ atoms at the appropriate geometry is partially compensated by the larger number of methyl hydrogen atoms in the cation. Bonds to fluorine atoms are less probable (0.6 bonds per cation) than HBs to O acceptors and engage mainly methyl hydrogens. HBs to the N atom of the anion appear in very small quantities (0.15 bonds/cation). The overall breakdown of donor/acceptor pairs in neat EMIM-TFSI is similar to that reported from AIMD simulations in our previous study,^33^ with the exception that in current simulations, the average numbers of HBs are smaller. We attribute this difference to the different DFT functionals used in calculations (Pade in ref (33), PBE in the current work). In the calculations with the Pade functional, maxima of the H_Im_–O_TFSI_, H_CH3_–O_TFSI_ and H_CH3_–F RDFs were shifted to lower distances by 0.15–0.25 Å, compared to PBE results. The number of HBs in the sample was therefore larger because the same value of the threshold distance was used in both works for HB detection.

In the systems containing water, H_2_O molecules tend to form HBs to oxygen atoms from TFSI anions. In the systems with 0.25 or 0.5 mole fraction of water, there is about 0.8 H_w_–O_TFSI_ HB per H_2_O molecule (the system *x* = 0.12 seems to be an outlier because of poor statistics when the simulation box contains only two water molecules). When the content of water increases, EMIM cations start to interact with H_2_O molecules (about 0.4 bond per cation in the *x* = 0.5 system). This competition between O_TFSI_ and O_w_ atoms for EMIM donors result in a small decrease of the number of EMIM–O_TFSI_ HBs. Nevertheless, the total number of HBs formed by the EMIM cation to oxygen atoms (TFSI or water) remains fairly constant and for all systems, it is in the range of 2.45–2.56 bonds/cation. We also note that the total number of HBs involving H atoms from the imidazolium ring and an O atom increases from 0.9 bonds/cation for neat IL to 1.0 in the *x* = 0.5 mixture.

With increasing water concentration, the probability of water–water hydrogen bonding also increases; at *x* = 0.5, there is 0.7 H_w_–O_w_ HB per water molecule. At lower water contents, there is also quite a substantial number of H_w_–N bonds (up to 0.25 bonds/H_2_O), disappearing at larger water concentration. The average number of HBs in the neat water simulated in AIMD is 1.91 bonds/molecule. In the IL/water mixtures, it is smaller and in the *x* = 0.5 system, there is about 1.61 HB/molecule formed by H_w_ atoms. The role of water as an HB acceptor is also reduced, with the H_2_O molecule accepting on average 1.15 hydrogen atoms. The average numbers of H atoms donated and accepted by water molecules in the *x* = 0.5 system are therefore 0.3 and 0.76 HB/H_2_O smaller than in the neat water. Finally, TFSI anions can accept hydrogens either from EMIM cations or water molecules, and the loss of EMIM-TFSI HBs is compensated by an increasing quantity of H_2_O–TFSI bonds. As a result, the average number of HBs per anion increases from 2.5 in neat IL to 3 in the *x* = 0.5 system. We can summarize the trends obtained from AIMD for IL/water systems as follows. In the mixture of solvents, water molecules form less HBs that in neat water. The total number of HBs formed by EMIM cations is almost unaffected, with interactions EMIM-H_2_O replacing EMIM-TFSI bonds at larger water concentrations. The number of bonds to TFSI anions increases because not all O atoms of the anion are used as HB acceptors in the neat IL, leaving the possibility to increase the number of bonds when additional hydrogen donors (water molecules) become available in the liquid.

### 3.2 IR Spectra

To give some information on the differences between the spectra calculated for individual replicas of the same system, we show in Figure S4 in the Supporting Information the IR spectra for *x* = 0 and *x* = 0.5, together with the averaged data. For neat IL, the differences are small. They increase in the IL/water mixture, especially in the region of water O–H vibrations. This was expected because for a small system size, the local structure of the liquid and the environments of water molecules can deviate from the average structure. In the main paper, we present therefore only the spectra averaged over all replicas.

**Figure 5:**
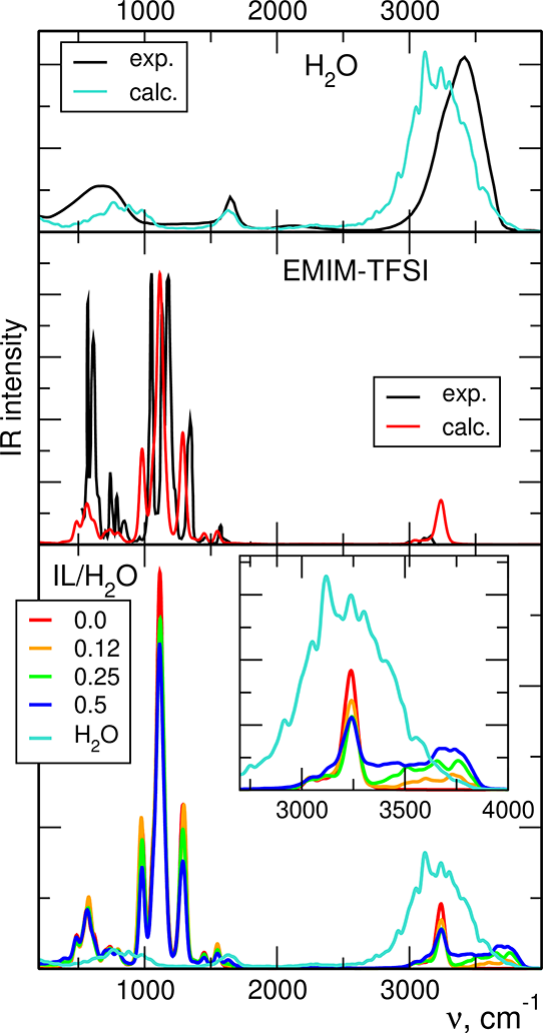
IR spectra calculated from AIMD DFT (PBE) trajectories for neat liquids and IL/water mixtures with increasing mole fraction of water.

The IR spectra of neat liquids obtained from our AIMD simulations applying PBE functional are shown in Figure 5, compared with experimental IR spectra of water^64^ and EMIM-TFSI.^65^ Although in both cases the overall agreement between calculations and experiment is quite satisfactory, there are some differences in the positions of bands. Computed frequencies of the H–O–H bending mode in water at 1600 cm^–1^ agree very well with measured data; on the other hand, the O–H stretching band above 3000 cm^–1^ is shifted by 200–250 cm^–1^ to lower energies with respect to the experiment. For EMIM-TFSI, the calculations predict correctly the shape of the spectrum; however, the part below 1500 cm^–1^ is shifted to lower frequencies by ca. 60 cm^–1^ and the intensity of the band calculated at 980 cm^–1^ is too low. There are also some differences in the region of C–H stretches, with a shift of computed spectrum to higher energies and increase of its intensity. These results are similar to the findings of a benchmarking study applying different functionals to model the vibrational spectra of liquid methanol.^66^ Most functionals (including PBE) underestimate the frequencies below 2000 cm^–1^. In the region above 2800 cm^–1^, frequencies of C–H and O–H stretches calculated in PBE functional are shifted upward and downward, respectively. Therefore, we consider the differences to the experiment observed in our results as acceptable because our primary task is to investigate the changes in the spectrum of the mixture of liquids with respect to a neat IL. Readers interested in the performance of different functionals are referred to the work of Kirchner et al.^66^

When the mole fraction of water in the system increases, the IR intensity of the bands related to IL vibrations decrease (bottom panel of Figure 5) due to decreasing concentration of ions in the solution. Much more interesting changes are visible in the region above 3000 cm^–1^ (inset in the Figure 5). There is a small intensity increase at frequencies just below the C–H band at 3240 cm^–1^. Simultaneously, the IR intensity between 3300 and 3800 cm^–1^ increases with water content, and a new maximum develops at about 3700–3750 cm^–1^. These trends agree very well with IR measurements of EMIM-TFSI/H_2_O^20^ and EMIM-BF_4_/H_2_O mixtures,^24^ in particular, with changes shown in the experimental spectra for increasing water mole fraction (Figure 7 of ref (20) and Figure 1a of ref (24)). The changes below the C–H band can be attributed to the shifts of C–H stretching frequencies in EMIM cations, which are in an environment different than that in neat IL. The intensity increase above the C–H band is due to O–H vibrations in H_2_O molecules. However, the new maximum appearing in Figure 5 (and in the experimental spectra in refs (20, 24)) is located above the main maximum observed for neat water. Therefore, the water molecules apparently are in an environment different than in the bulk water, with a smaller degree of H_2_O–H_2_O hydrogen bonding.

To investigate in more detail the relation between vibrational frequency of a given X–H stretch and the local structure of the liquid, we calculated Fourier transforms (FTs) of all C_1_–H and O–H bond lengths and H–O–H angles in the sample. For a clearer presentation, the obtained frequencies were then represented by Gaussian curves with σ = 5 cm^–1^. In the Supporting Information (Figure S5), we show the FTs averaged over all EMIM ions/water molecules in the system. The averaged data exhibit similar changes to those observed in calculated IR spectra when the water fraction is increased. More information can be retrieved from nonaveraged FTs analyzed for individual ions or molecules.

**Figure 6:**
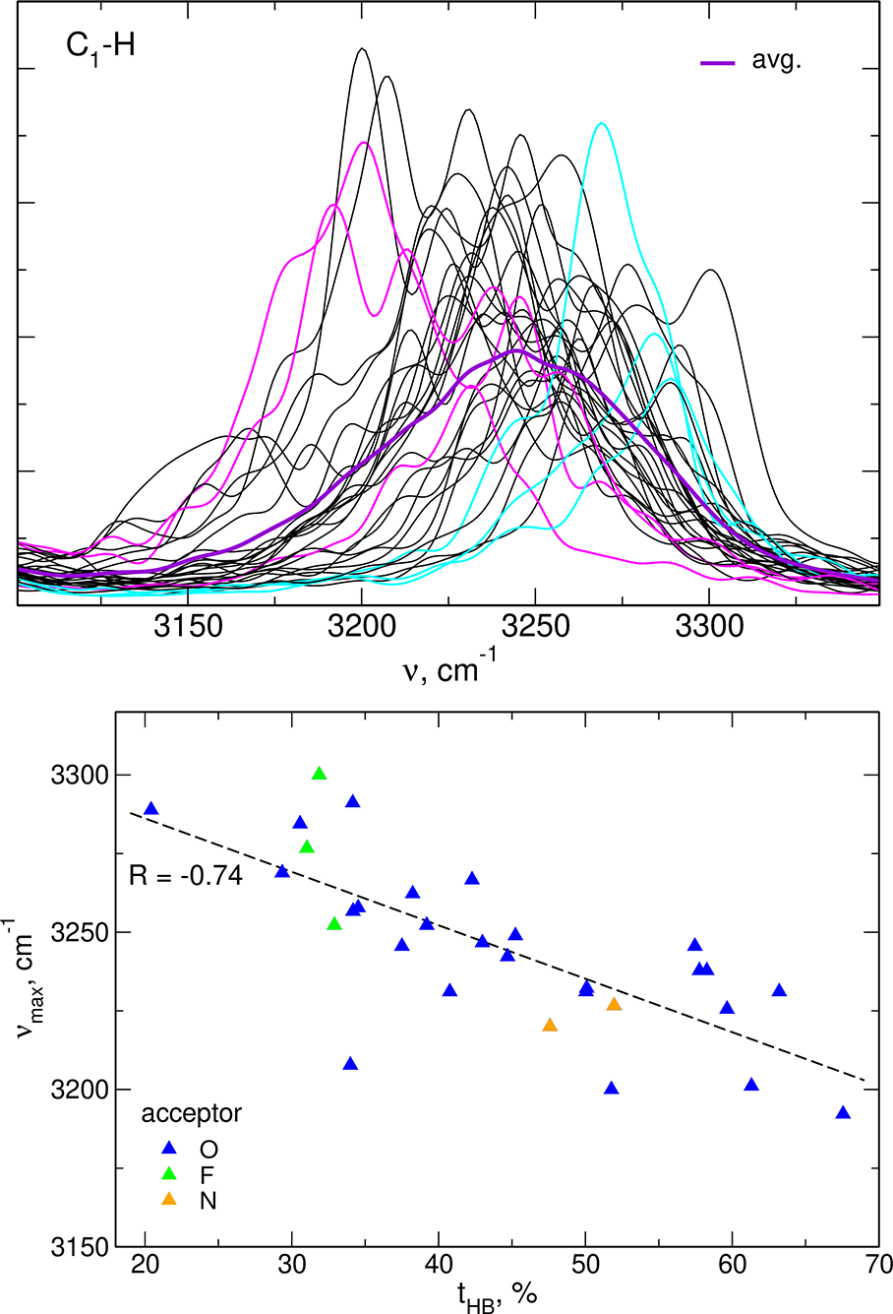
Fourier transforms of C_1_–H bond lengths in neat EMIM-TFSI (upper panel); positions of the maxima in FTs of bond lengths vs the time of HB formation (lower panel).

A sample plot of Fourier-transformed C_1_–H bond lengths of all EMIM cations in the neat EMIM-TFSI liquid is presented in the upper panel of Figure 6. As readily seen, frequencies of individual bonds vary in an interval of about 200 cm^–1^, with the average frequency (purple line) of 3250 cm^–1^. We selected three EMIM ions, which spent most time forming the HB from C_1_–H group to the TFSI anion (shown in magenta in Figure 6), and the three ions with the shortest time of HB formation (cyan curves). Frequencies of C–H vibrations involved in HB formation are shifted below the average frequency, whereas frequencies of “free” C–H bonds (that is, participating in HBs for only small part of the time of simulation) are higher than the average. We should note that in a homogeneous system, for a sufficiently long simulation time, all bonds will be on average in the same environment; therefore, all frequencies should be close to the average over the whole sample. Yet, in a given moment of time, the local environments of C–H bonds may be different, and this difference can be observed when the data are analyzed in a sufficiently narrow time window (that is, for time intervals shorter than the mean time between environment changes), as it is the case of our simulation.

In the lower panel of Figure 6, we show at which frequency a maximum in Fourier-transformed C_1_–H length appears, depending on the average percentage of time spent on formation of C_1_–H···O_TFSI_ HBs. These two parameters are correlated; the frequency of the C–H stretch decreases when the H atom forms a HB to the anion. The acceptor in the majority of these HBs is an oxygen atom; therefore, the statistics from the small system is not sufficient to make an analysis for different acceptors; nevertheless, the plot suggests that the bonds formed to F atoms (green symbols in Figure 6) lead to smaller decrease of the C–H frequency.

**Figure 7:**
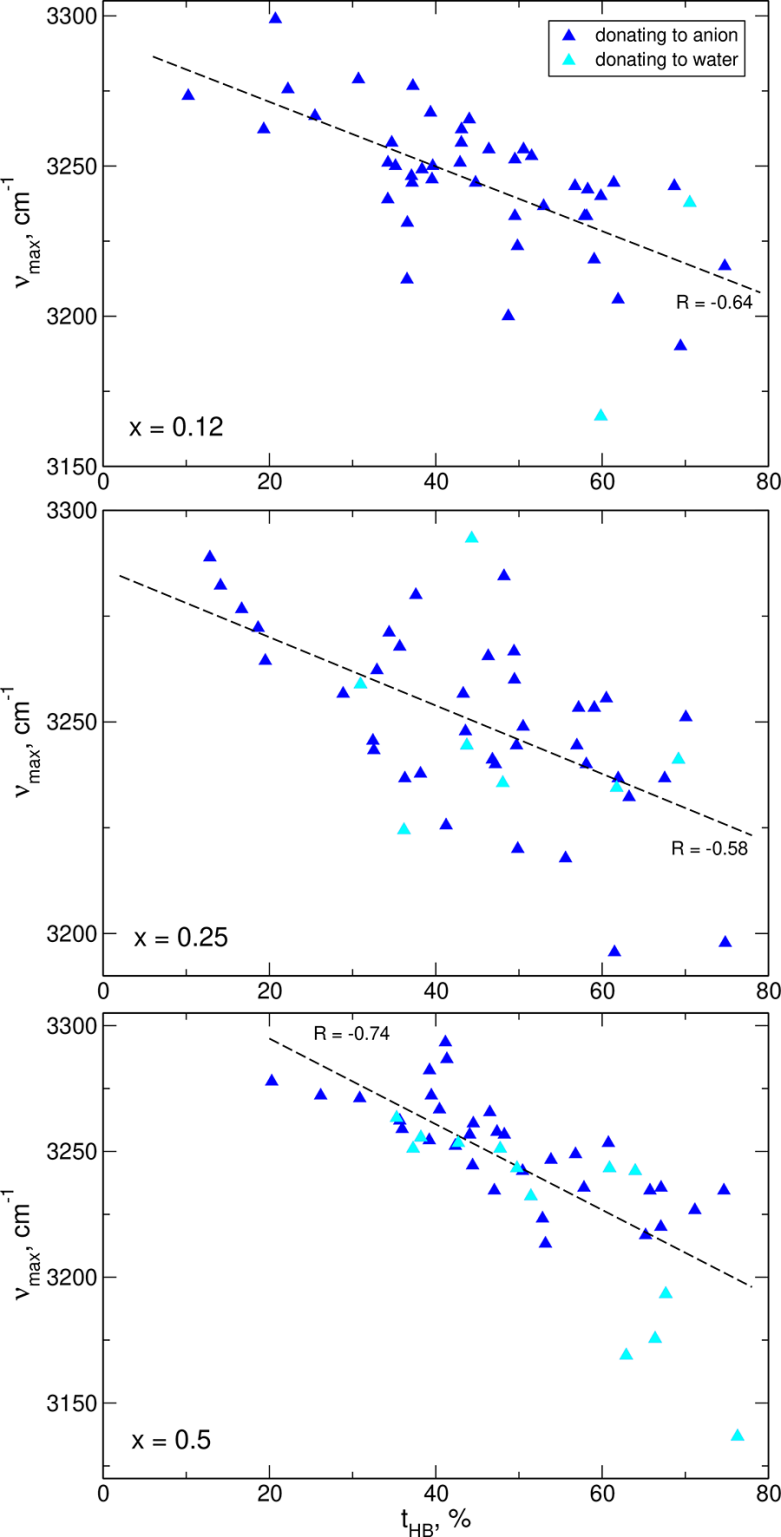
Positions of the maxima in FTs of C_1_–H bond lengths vs the time of HB formation for IL/water mixtures.

Similar analysis was performed for C_1_–H frequencies resulting from FTs of bond lengths in the systems containing water molecules. The combined results are shown in Figure 7. The time of HB formation includes equally the bonds to TFSI anions and water molecules; in the plots, we marked the cations according to the acceptor of the H atom. In all three systems, the correlation is similar to that presented for the neat IL: hydrogen bonding decreases the frequency of the stretching vibration. There is no obvious correlation with the type of acceptor (TFSI or water), perhaps with the exception of the *x* = 0.5 system in which the most red-shifted vibrations engage water molecules. Again, the sizes of systems treated in AIMD are too small to collect more data and to improve the statistics.

With the results displayed in Figures 6 and 7, we can conclude that the increase of the IR intensity observed below the band at 3250 cm^–1^ in water-containing samples is related to changes in hydrogen bonding of H atoms from EMIM cations. Statistics of HBs (Section 3.1) shows the increase of the probability of HB formation by imidazolium hydrogens with increasing water fraction. As a result, more C–H stretches are affected by hydrogen bonding and with an increased average time of HB formation, the red-shifts of frequencies are larger, leading to the changes observed in calculated/measured spectra.

To analyze the changes in the spectra above 3300 cm^–1^, we performed the FT-based analysis of O–H vibrations in water molecules. It is well known that frequencies of these stretches are sensitive to the configuration of HBs in which given water molecule participates. Results of fitting Raman spectrum of water in the region of O–H stretches^67^ showed that the “free” O–H bonds (that is, not being a donor of a HB) appear at the highest frequencies (∼ 3600 cm^–1^). The frequencies of O–H bonds involved in an HB decrease in the order DDA, DA, DDAA, and DAA, depending on the HB configuration of a given molecule.^67^ Here, D and A denote that the H_2_O molecule serves as donor and acceptor of a HB, respectively, e.g., in the DDA configuration the molecule is an acceptor of one hydrogen atom from another molecule and simultaneously donates both hydrogen atoms to two other molecules. The frequency fitted to the DDAA configuration (“tetrahedral” water) was ∼3200 cm^–1^, and the other main contribution at ∼3400 cm^–1^ was assigned to DA configuration.^67^

**Figure 8:**
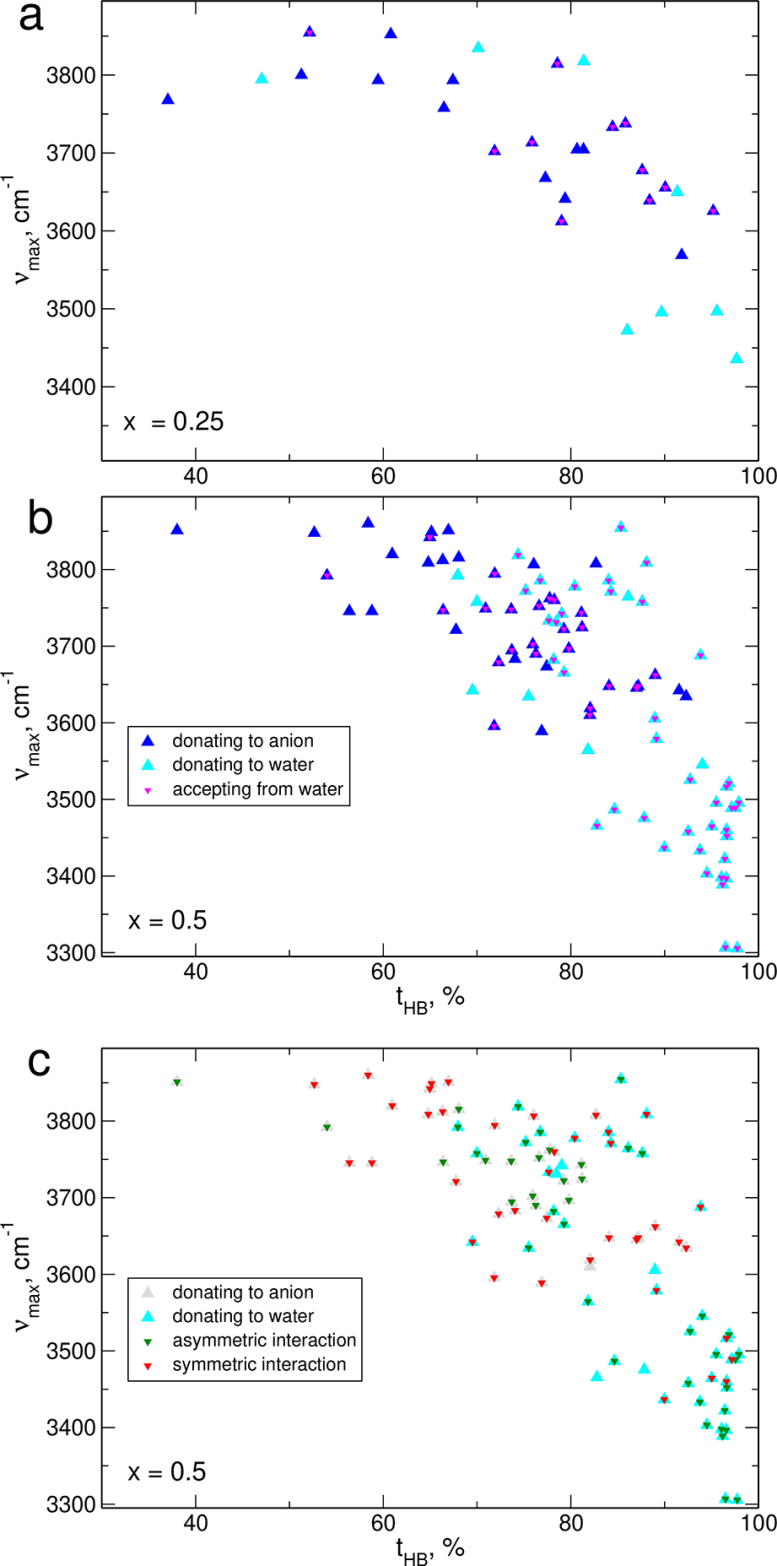
Positions of the maxima in FTs of O–H bond lengths vs the time of HB formation for IL/water mixtures with *x* = 0.25 (a); *x* = 0.5 (b); alternative labeling of data for *x* = 0.5 (c).

In Figure 8a,b we show the frequencies of O–H stretches vs percentage of the time of HB formation for all replicas of systems with water mole fraction *x* = 0.25 and 0.5. We marked in the plot whether the HB formed by the O–H hydrogen is mainly to TFSI or to water and whether the molecule at hand is an acceptor of a HB from another H_2_O molecule. The increased time of HB formation generally results in a decrease of O–H stretching frequency. One can note, however, that the configuration of HBs of given molecule is another factor modifying the vibrational frequencies. Donation of H atom to TFSI anion results in smaller red-shifts than the donation to water molecule. The O–H frequency is further shifted to lower energies when the H_2_O molecule is additionally an acceptor of hydrogen atom from another water molecule. Therefore, the lowest frequencies (3500–3300 cm^–1^) are computed for O–H vibrations in H_2_O molecules being simultaneously H-acceptors and H-donors from/to other water molecules, that is, for the molecules that are in a configuration of HBs most close to the situation in a bulk water. The number of such water molecules in an IL/water mixture is, however, limited. Some H_2_O molecules are not involved at all in interactions with other water molecules and serve only as H-donors to TFSI anions. In such cases, the O–H frequency is usually computed between 3850 and 3700 cm^–1^. The frequency is lowered to 3800–3600 cm^–1^ if the water molecule donating to TFSI anion becomes also an HB acceptor from another H_2_O molecule. Finally, in molecules not being an HB acceptor from water but donating the hydrogen to a water molecule, the O–H stretching frequency is in the range 3800–3500 cm^–1^. Therefore, when the water content in the IL increases, most of water molecules are in hydrogen bonding environment different than in the bulk water. These molecules (a) form an HB only for short time or (b) donate H atom to the anion of the IL or/and (c) are not an acceptor of H atom from another water molecule. Frequencies of corresponding O–H stretches are on average higher that in the bulk water, and therefore, in the calculated (Figure 5) and in the measured IR spectra,^20,24^ a new band appears above the IR band of the bulk H_2_O.

In Figure 8c, we present an alternative analysis of HB donor–acceptor structure for *x* = 0.5 data, based on the symmetry of the solvating environment.^24^ In addition to information on the HB acceptor, we color-coded the distinction between symmetric (both O–H groups form HBs to same type of acceptor for a similar time) and asymmetric interactions (the acceptors for the two O–H bonds are different or there is a substantial difference in time spent on HB formation). According to Scheme 1 in ref (24), frequencies of O–H stretch depend on the solvation environment as follows: ν_ab_ < ν_s_ < ν_af_, where ν_s_ is the frequency in symmetric environment and ν_ab_ and ν_af_ stand for the frequency of “bound” and “free” O–H groups, respectively, in an asymmetric configuration.

Indeed, the lowest frequencies are computed for O–H groups donating to water in an asymmetric environment (that is, in the cases where the other O–H group is “free” or donating to the anion). Frequencies for symmetric interactions are located at higher values. We should expect that the highest frequencies are obtained for O–H groups interacting asymmetrically with anion for short time (ν_af_ frequency). Unfortunately, only one such O–H vibration is visible in Figure 8c, but its frequency is among the highest calculated. Therefore, the general trend discussed in ref (24) seems to exists in our data, but for detailed analysis, a better statistics collected from larger systems and longer simulation times would be required.

### 3.3 Performance of DFTB Methodology

**Figure 9:**
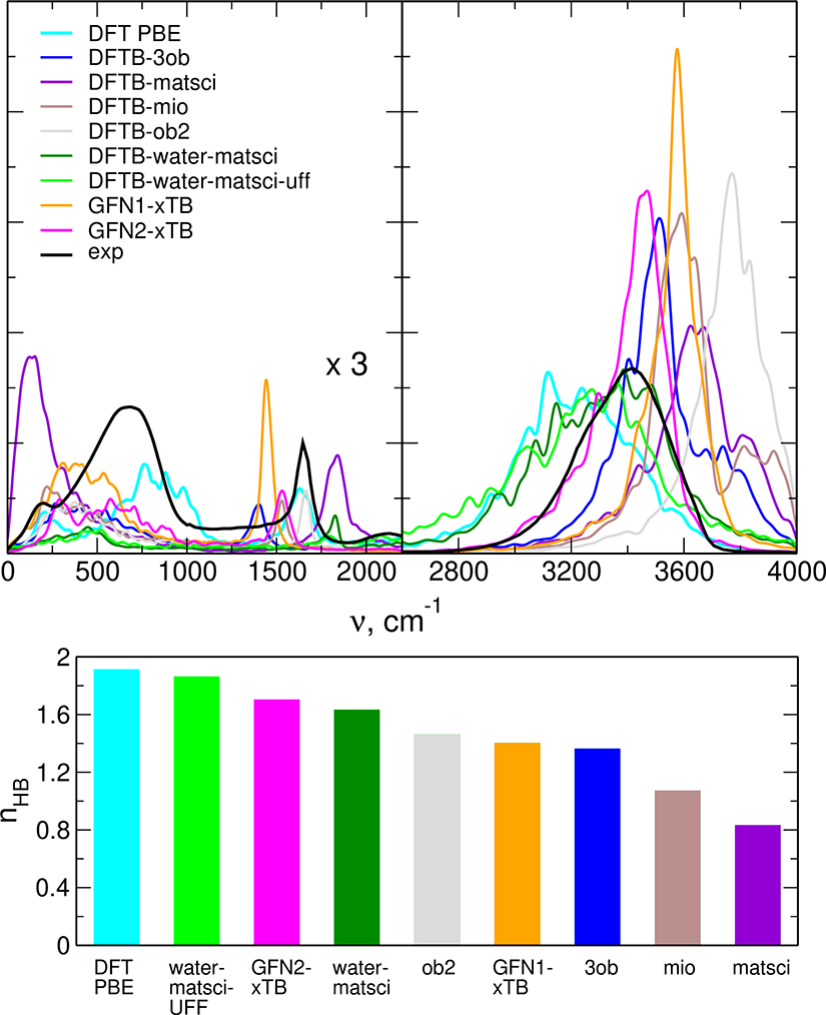
IR spectra calculated for bulk water in different DFTB parameterizations (top); average number of HBs per water molecule (bottom).

We started the tests of DFTB methodology with bulk water, for which many ready-to-use parameterizations are available. In the top panels of Figure 9, we present the calculated IR spectra compared to the experimental data and the results of AIMD simulations with the PBE functional. The performance of DFTB in the low-frequency part of the spectrum is rather poor; therefore, we focus on the region of O–H stretches. The two parameterizations designed specifically for water, *water-matsci* and *water-matsci-uff*, give the best agreement with experimental data and with the DFT result. Next to these two are GFN2-xTB, *3ob*, and GFN1-xTB. In Figure S6 in the Supporting Information, we show the O–O RDFs in water calculated for different parameterizations, and the best agreement with the DFT result is obtained for those parameterizations that also yield the best IR spectra.

We also tried to relate the quality of spectrum reproduction to the number of HBs in the simulated structures. In the lower panel of Figure 9, we show the average number of HBs per water molecule. Closest to the DFT data is the *water-matsci-uff* parameterization, and also GFN2-xTB and *water-matsci* performed relatively well. Nevertheless, there is no direct relation of the number of HBs to the quality of the spectrum, for example, *3ob* performs better than GFN1-xTB and *ob2* in the reproduction of the spectrum, although it is worse in predicting the number of HBs. A possible origin of this behavior may be seen in Figure S7 in the Supporting Information, showing the SDFs of O_w_ atoms around the H_2_O molecule—the *3ob* parameterization gives the SDF closer to the tetrahedral structure of water. We can summarize that the best description of the structure of water is obtained for *water-matsci* and *water-matsci-uff* parameterizations, which is not surprising, as they were designed for this purpose. We confirmed that this good reproduction of the structure of the liquid results also in good performance in simulations of IR spectrum. The other well performing method is the general GFN2-xTB parameterization.

**Figure 10:**
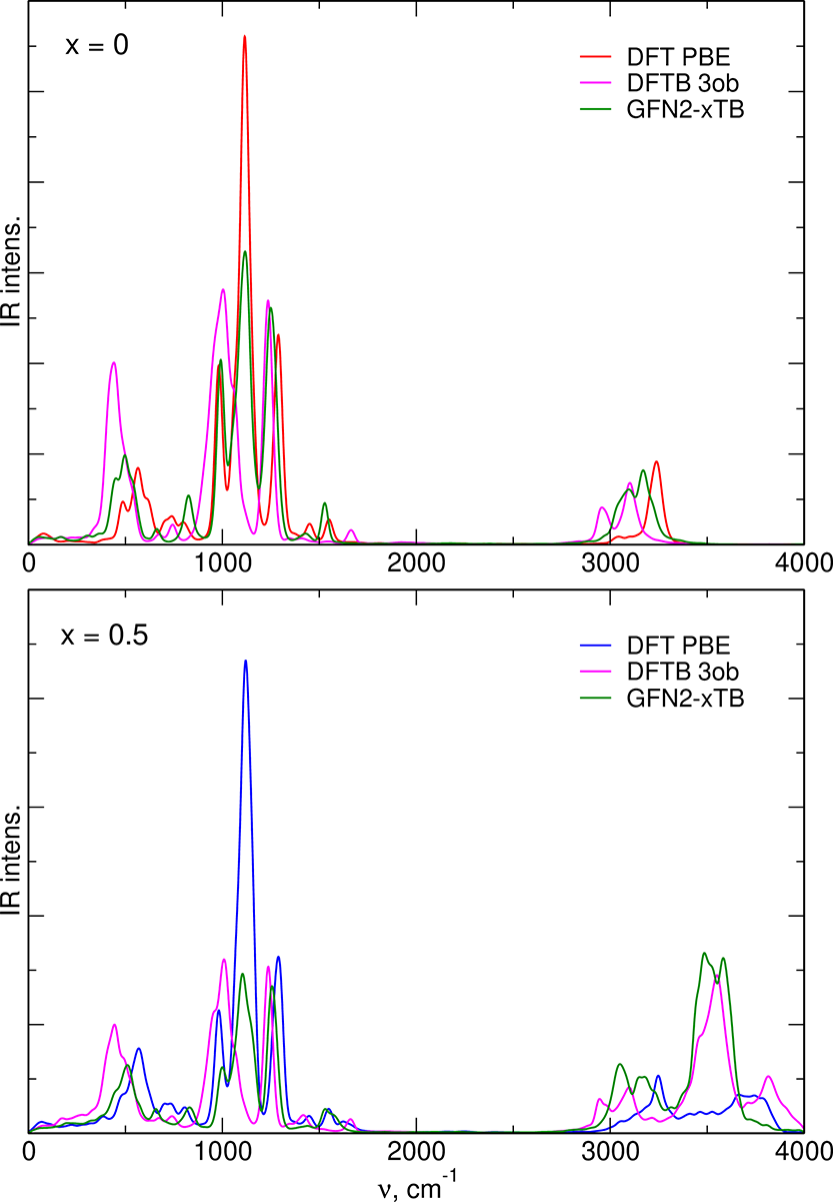
IR spectra of neat IL and the mixture with water obtained from MD based on PBE DFT or two DFTB parameterizations.

The IR spectra of neat EMIM-TFSI and the *x* = 0.5 solution are displayed in Figure 10 with the results of DFT PBE calculations shown for comparison. Both in neat IL and in the mixture with water, the *3ob* parameterization predicts two main IR bands in the range of 1000–1300 cm^–1^, instead of three calculated in DFT. The GFN2-xTB correctly reproduces the number of bands, and also their frequencies are closer to DFT PBE results. For neat IL in the region above 3000 cm^–1^, the results are similar, with *3ob* yielding too low frequencies and the xTB result closer (but not in exact agreement) to DFT PBE. In the system with water, both DFTB approaches differ noticeably from DFT. In both cases, an intense band appears at 3500 cm^–1^. At the high frequency part of the spectrum, there is an increased IR intensity in *3ob* results; on the other hand, the intensity of GFN2-xTB spectrum above 3700 cm^–1^ is small.

To get some information on the structure of the liquid, we calculated distribution functions for the *x* = 0.5 system (Figures S8–S10 in the Supporting Information). The height of the maxima in H–O_TFSI_ RDFs obtained in *3ob* simulations is reduced. On the other hand, the H_CH3_–O_w_ maximum in the GFN2-xTB is much larger than in DFT results, suggesting a possible increase in the number of hydrogen bonds between methyl groups and water molecules. The CDFs for GFN2-xTB in Figure S9 compared to DFT data in Figure 2 also show an increased probability of occurrence of H_CH3_–O_w_ and H_CH3_–O_TFSI_ configurations suitable for HB formation. The SDFs in Figure S10 suggest that the TFSI and water O atoms are more evenly distributed near EMIM cations, and the probability is concentrated not only close to H_Im_ hydrogen atoms.

**Figure 11:**
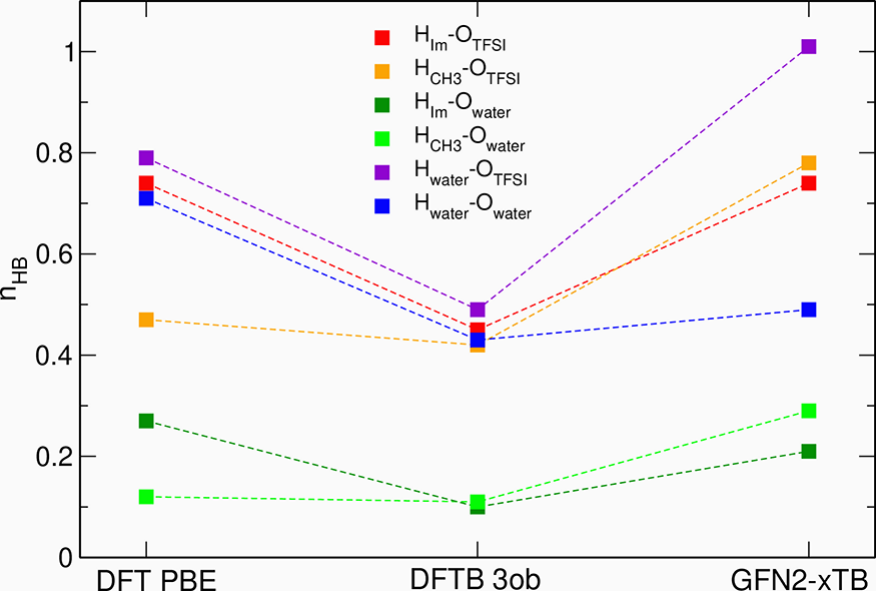
Average numbers of the most abundant hydrogen bonds obtained for *x* = 0.5 from MD simulations based on DFT or DFTB. Lines are only to guide the eye.

The average numbers of the most abundant types of HBs are compared for three simulation methods in Figure 11 (*x* = 0.5) and Figures S11 and S12 (full statistics for neat IL and *x* = 0.5 systems). In *3ob* results, the number of all main types of HBs is reduced and apparently this method predicts weaker hydrogen bonding ability of EMIM-TFSI/water. This observation may help to explain the maximum at ∼3800 cm^–1^ in the *3ob* IR spectrum—there are more “free” O–H bonds in water molecules contributing to this part of the spectrum. As expected from distribution functions, in the structures obtained from GFN2-xTB, there are more H_CH3_–O_TFSI_ and H_CH3_–O_w_ bonds. In the statistics of HBs with water as a donor, the ratio of TFSI and H_2_O acceptors is changed in favor of the anions of the IL. Both *3ob* and GFN2-xTB methods result in distribution of HBs different from that obtained at the DFT PBE level. Although it is not clear why the IR band at 3500 cm^–1^ develops in both cases, the simulated IR spectra apparently do not agree with measured data^20,24^ (in contrast to PBE results); therefore, we may conclude that the description of the HB network in both tested DFTB approaches is not good enough to obtain a satisfactory reproduction of experimental spectra. It could be probably improved with a redesigned parameterization, like the good performance of tailored parameters sets that was found in calculations of the IR spectrum of bulk water.

## 4 Conclusions

We performed DFT-based AIMD simulations for mixtures of EMIM-TFSI ionic liquid and an increasing amount of water. Analysis of hydrogen bonding interactions shows that the most abundant are the HBs with imidazolium carbon atoms as donors and oxygen atoms as acceptors. The average number of HBs formed by EMIM cations is barely affected by the presence of water, and the number of HBs with TFSI acceptors increases with the mole fraction of H_2_O.

The IR spectra calculated from the AIMD trajectories are in good agreement with available experimental data for EMIM-TFSI or EMIM-BF_4_ mixtures with water. The effects of increased water content are well reproduced in the spectrum above 3000 cm^–1^. Using the FT analysis of bond lengths in individual ions or molecules, we related the local environment and formation of HBs to the changes observed in the spectrum. In this way, we confirmed that the increasing IR intensity in the range of water O–H vibrations is due to H_2_O molecules with HB patterns different than in neat water, either with “free” O–H groups or forming an HB to the anion of the IL.

From several DFTB parameterizations tested for neat water, the best performing in the reproduction of IR spectra were two parameter sets designed specifically for water and also the general-purpose GFN2-xTB method. However, neither of the two parameterizations used for IL/water mixtures were able to capture the changes induced in the IR spectrum by the presence of water; this shortfall of DFTB could be related to improper description of hydrogen bonding between IL and H_2_O. We concluded therefore that the DFT AIMD simulations are very promising for investigations of the structure and vibrational spectra of IL/water systems and for explaining spectral shifts caused by HBs. On the other hand, the application of DFTB methodology for these purposes would need an improvement of parameterization, tailored to a particular system. The other possibility, which we intend to exploit in the future, is the use of machine learning to obtain potential energy surfaces for MD simulations, as done recently, e.g., for bulk water.^68^

## Supporting Information Available

Distribution functions for neat IL, IR spectra for individual trajectories, averaged FTs of bond lengths, O–O distribution functions for bulk water in different DFTB parameterizations, distribution functions obtained in GFN2-xTB, statistics of HBs for neat IL in different DFTB parameterizations, and sample input files (PDF)

## References

[ref1] Fumino K.; Peppel T.; Geppert-Rybczyńska M.; Zaitsau D. H.; Lehmann J. K.; Verevkin S. P.; Köckerling M.; Ludwig R. The Influence of Hydrogen Bonding on the Physical Properties of Ionic Liquids. Phys. Chem. Chem. Phys. 2011, 13, 14064–14075. 10.1039/c1cp20732f. 21666914

[ref2] Hunt P. A.; Ashworth C. R.; Matthews R. P. Hydrogen Bonding in Ionic Liquids. Chem. Soc. Rev. 2015, 44, 1257–1288. 10.1039/C4CS00278D. 25582457

[ref3] Hayes R.; Warr G. G.; Atkin R. Structure and Nanostructure in Ionic Liquids. Chem. Rev. 2015, 115, 6357–6426. 10.1021/cr500411q. 26028184

[ref4] Wang Y.-L.; Li B.; Sarman S.; Mocci F.; Lu Z.-Y.; Yuan J.; Laaksonen A.; Fayer M. D. Microstructural and Dynamical Heterogeneities in Ionic Liquids. Chem. Rev. 2020, 120, 5798–5877. 10.1021/acs.chemrev.9b00693. 32292036 PMC7349628

[ref5] Abe H. Phase Variety in Ionic Liquids: Hydrogen Bonding and Molecular Conformations. J. Mol. Liq. 2021, 332, 115189 10.1016/j.molliq.2020.115189.

[ref6] Seddon K. R.; Stark A.; Torres M. J. Influence of Chloride, Water, and Organic Solvents on the Physical Properties of Ionic Liquids. Pure Appl. Chem. 2000, 72, 2275–2287. 10.1351/pac200072122275.

[ref7] Porter A. R.; Liem S. Y.; Popelier P. L. A. Room Temperature Ionic Liquids Containing Low Water Concentrations-A Molecular Dynamics Study. Phys. Chem. Chem. Phys. 2008, 10, 4240–4248. 10.1039/b718011j. 18633544

[ref8] Moreno M.; Castiglione F.; Mele A.; Pasqui C.; Raos G. Interaction of Water with the Model Ionic Liquid [bmim][BF4]: Molecular Dynamics Simulations and Comparison with NMR Data. J. Phys. Chem. B 2008, 112, 7826–7836. 10.1021/jp800383g. 18537287

[ref9] Méndez-Morales T.; Carrete J.; Cabeza Ó.; Gallego L. J.; Varela L. M. Molecular Dynamics Simulation of the Structure and Dynamics of Water-1-alkyl-3-methylimidazolium Ionic Liquid Mixtures. J. Phys. Chem. B 2011, 115, 6995–7008. 10.1021/jp202692g. 21561120

[ref10] Zhang Q.-G.; Wang N.-N.; Wang S.-L.; Yu Z.-W. Hydrogen Bonding Behaviors of Binary Systems Containing the Ionic Liquid 1-Butyl-3-methylimidazolium Trifluoroacetate and Water/Methanol. J. Phys. Chem. B 2011, 115, 11127–11136. 10.1021/jp204305g. 21842910

[ref11] Zhong X.; Fan Z.; Liu Z.; Cao D. Local Structure Evolution and its Connection to Thermodynamic and Transport Properties of 1-Butyl-3-methylimidazolium Tetrafluoroborate and Water Mixtures by Molecular Dynamics Simulations. J. Phys. Chem. B 2012, 116, 3249–3263. 10.1021/jp3001543. 22352309

[ref12] Martins V. L.; Nicolau B. G.; Urahata S. M.; Ribeiro M. C. C.; Torresi R. M. Influence of the Water Content on the Structure and Physicochemical Properties of an Ionic Liquid and Its Li^+^ Mixture. J. Phys. Chem. B 2013, 117, 8782–8792. 10.1021/jp312839z. 23815781

[ref13] Dziubinska-Kühn K.; Croese J.; Pupier M.; Matysik J.; Viger-Gravel J.; Karg B.; Kowalska M. Structural Analysis of Water in Ionic Liquid Domains – A Low Pressure Study. J. Mol. Liq. 2021, 334, 116447 10.1016/j.molliq.2021.116447.

[ref14] Gliege M. E.; Lin W. J.; Xu Y.; Chen M.-T.; Whitney C.; Gunckel R.; Dai L. Molecular Dynamics Insight into the Role of Water Molecules in Ionic Liquid Mixtures of 1-Butyl-3-methylimidazolium Iodide and Ethylammonium Nitrate. J. Phys. Chem. B 2022, 126, 1115–1124. 10.1021/acs.jpcb.1c05595. 35107286

[ref15] Cammarata L.; Kazarian S. G.; Salter P. A.; Welton T. Molecular States of Water in Room Temperature Ionic Liquids. Phys. Chem. Chem. Phys. 2001, 3, 5192–5200. 10.1039/b106900d.

[ref16] Köddermann T.; Wertz C.; Heintz A.; Ludwig R. The Association of Water in Ionic Liquids: A Reliable Measure of Polarity. Angew. Chem., Int. Ed. 2006, 45, 3697–3702. 10.1002/anie.200504471. 16637092

[ref17] Takamuku T.; Kyoshoin Y.; Shimomura T.; Kittaka S.; Yamaguchi T. Effect of Water on Structure of Hydrophilic Imidazolium-Based Ionic Liquid. J. Phys. Chem. B 2009, 113, 10817–10824. 10.1021/jp9042667. 19603782

[ref18] Danten Y.; Cabaço M. I.; Besnard M. Interaction of Water Diluted in 1-Butyl-3-methyl Imidazolium Ionic Liquids by Vibrational Spectroscopy Modeling. J. Mol. Liq. 2010, 153, 57–66. 10.1016/j.molliq.2009.07.001.

[ref19] Cha S.; Ao M.; Sung W.; Moon B.; Ahlström B.; Johansson P.; Ouchi Y.; Kim D. Structures of Ionic Liquid–Water Mixtures Investigated by IR and NMR Spectroscopy. Phys. Chem. Chem. Phys. 2014, 16, 9591–9601. 10.1039/C4CP00589A. 24728507

[ref20] Yaghini N.; Pitawala J.; Matic A.; Martinelli A. Effect of Water on the Local Structure and Phase Behavior of Imidazolium-Based Protic Ionic Liquids. J. Phys. Chem. B 2015, 119, 1611–1622. 10.1021/jp510691e. 25548901

[ref21] Kundu K.; Chandra G. K.; Umapathy S.; Kiefer J. Spectroscopic and Computational Insights into the Ion–Solvent Interactions in Hydrated Aprotic and Protic Ionic Liquids. Phys. Chem. Chem. Phys. 2019, 21, 20791–20804. 10.1039/C9CP03670A. 31513201

[ref22] Yoshimura Y.; Mori T.; Mori T.; Hattori S.; Kaneko K.; Ueda J.; Takekiyo T.; Shimizu A. Insights into the Local Structures of Water in 1-Butyl-3-methylimidazolium Iodide. J. Mol. Liq. 2020, 319, 114152 10.1016/j.molliq.2020.114152.

[ref23] Biswas A.; Mallik B. S. Vibrational Spectral Dynamics and Ion-Probe Interactions of the Hydrogen-Bonded Liquids in 1-Ethyl-3-methylimidazolium Bis(trifluoromethylsulfonyl)imide. Chem. Phys. 2022, 559, 111519 10.1016/j.chemphys.2022.111519.

[ref24] Mukherjee K.; Palchowdhury S.; Maroncelli M. OH Stretching and Libration Bands of Solitary Water in Ionic Liquids and Dipolar Solvents Share a Single Dependence on Solvent Polarity. J. Phys. Chem. B 2022, 126, 4584–4598. 10.1021/acs.jpcb.2c02445. 35687693

[ref25] Bottari C.; Almásy L.; Rossi B.; Bracco B.; Paolantoni M.; Mele A. Interfacial Water and Microheterogeneity in Aqueous Solutions of Ionic Liquids. J. Phys. Chem. B 2022, 126, 4299–4308. 10.1021/acs.jpcb.1c10961. 35649236 PMC9207890

[ref26] Voss J. M.; Marsh B. M.; Zhou J.; Garand E. Interaction Between Ionic Liquid Cation and Water: Infrared Predissociation Study of [bmim]^+^ · (H_2_O)_n_ Clusters. Phys. Chem. Chem. Phys. 2016, 18, 18905–18913. 10.1039/C6CP02730J. 27353528

[ref27] Singh D. K.; Donfack P.; Rathke B.; Kiefer J.; Materny A. Interplay of Different Moieties in the Binary System 1-Ethyl-3-methylimidazolium Trifluoromethanesulfonate/Water Studied by Raman Spectroscopy and Density Functional Theory Calculations. J. Phys. Chem. B 2019, 123, 4004–4016. 10.1021/acs.jpcb.9b00066. 30986056

[ref28] Shyama M.; Lakshmipathi S. Water Confined (H_2_O)_n=1–10_ Amino Acid-Based Ionic Liquids – A DFT Study on the Bonding, Energetics and IR Spectra. J. Mol. Liq. 2020, 304, 112720 10.1016/j.molliq.2020.112720.

[ref29] Mondal A.; Balasubramanian S. Vibrational Signatures of Cation–Anion Hydrogen Bonding in Ionic Liquids: A Periodic Density Functional Theory and Molecular Dynamics Study. J. Phys. Chem. B 2015, 119, 1994–2002. 10.1021/jp5113679. 25587624

[ref30] Zentel T.; Kühn O. Hydrogen Bonding in the Protic Ionic Liquid Triethylammonium Nitrate Explored by Density Functional Tight Binding Simulations. J. Chem. Phys. 2016, 145, 234504 10.1063/1.4972006. 27984873

[ref31] Eilmes A.; Kubisiak P.; Brela M. Explicit Solvent Modeling of IR and UV–Vis Spectra of 1-Ethyl-3-methylimidazolium Bis(trifluoromethylsulfonyl)imide Ionic Liquid. J. Phys. Chem. B 2016, 120, 11026–11034. 10.1021/acs.jpcb.6b07994. 27696848

[ref32] Thomas M.; Brehm M.; Hollóczki O.; Kelemen Z.; Nyulászi L.; Pasinszki T.; Kirchner B. Simulating the Vibrational Spectra of Ionic Liquid Systems: 1-Ethyl-3-methylimidazolium Acetate and its Mixtures. J. Chem. Phys. 2014, 141, 024510 10.1063/1.4887082. 25028030

[ref33] Brela M. Z.; Kubisiak P.; Eilmes A. Understanding the Structure of the Hydrogen Bond Network and Its Influence on Vibrational Spectra in a Prototypical Aprotic Ionic Liquid. J. Phys. Chem. B 2018, 122, 9527–9537. 10.1021/acs.jpcb.8b05839. 30239203

[ref34] Elstner M.; Porezag D.; Jungnickel G.; Elsner J.; Haugk M.; Frauenheim T.; Suhai S.; Seifert G. Self-Consistent-Charge Density-Functional Tight-Binding Method for Simulations of Complex Materials Properties. Phys. Rev. B 1998, 58, 7260–7268. 10.1103/PhysRevB.58.7260.

[ref35] Gaus M.; Cui Q.; Elstner M. DFTB3: Extension of the Self-Consistent-Charge Density-Functional Tight-Binding Method (SCC-DFTB). J. Chem. Theory Comput. 2011, 7, 931–948. 10.1021/ct100684s. PMC3509502 23204947

[ref36] Addicoat M. A.; Stefanovic R.; Webber G. B.; Atkin R.; Page A. J. Assessment of the Density Functional Tight Binding Method for Protic Ionic Liquids. J. Chem. Theory Comput. 2014, 10, 4633–4643. 10.1021/ct500394t. 25328497 PMC4196743

[ref37] Zentel T.; Kühn O. Properties of Hydrogen Bonds in the Protic Ionic Liquid Ethylammonium Nitrate. Theor. Chem. Acc. 2017, 136, 87 10.1007/s00214-017-2119-6.

[ref38] Okoshi M.; Chou C.-P.; Nakai H. Theoretical Analysis of Carrier Ion Diffusion in Superconcentrated Electrolyte Solutions for Sodium-Ion Batteries. J. Phys. Chem. B 2018, 122, 2600–2609. 10.1021/acs.jpcb.7b10589. 29433319

[ref39] Martínez L.; Andrade R.; Birgin E. G.; Martínez J. M. Packmol: A Package for Building Initial Configurations for Molecular Dynamics Simulations. J. Comput. Chem. 2009, 30, 2157–2164. 10.1002/jcc.21224. 19229944

[ref40] Phillips J. C.; Braun R.; Wang W.; Gumbart J.; Tajkhorshid E.; Villa E.; Chipot C.; Skeel R. D.; Kalé L.; Schulten K. Scalable Molecular Dynamics with NAMD. J. Comput. Chem. 2005, 26, 1781–1802. 10.1002/jcc.20289. 16222654 PMC2486339

[ref41] Kubisiak P.; Eilmes A. Molecular Dynamics Simulations of Ionic Liquid Based Electrolytes for Na-Ion Batteries: Effects of Force Field. J. Phys. Chem. B 2017, 121, 9957–9968. 10.1021/acs.jpcb.7b08258. 28976751

[ref42] Canongia Lopes J. N.; Deschamps J.; Pádua A. A. H. Modeling Ionic Liquids Using a Systematic All-Atom Force Field. J. Phys. Chem. B 2004, 108, 2038–2047. 10.1021/jp0362133.

[ref43] Köddermann T.; Paschek D.; Ludwig R. Molecular Dynamic Simulations of Ionic Liquids: A Reliable Description of Structure, Thermodynamics and Dynamics. ChemPhysChem 2007, 8, 2464–2470. 10.1002/cphc.200700552. 17943710

[ref44] Jorgensen W. L.; Chandrasekhar J.; Madura J. D.; Impey R. W.; Klein M. L. Comparison of Simple Potential Functions for Simulating Liquid Water. J. Chem. Phys. 1983, 79, 926–935. 10.1063/1.445869.

[ref45] Feller S. E.; Zhang Y.; Pastor R. W.; Brooks B. R. Constant Pressure Molecular Dynamics Simulation: The Langevin Piston Method. J. Chem. Phys. 1995, 103, 4613–4621. 10.1063/1.470648.

[ref46] Martyna G. J.; Tobias D. J.; Klein M. L. Constant Pressure Molecular Dynamics Algorithms. J. Chem. Phys. 1994, 101, 4177–4189. 10.1063/1.467468.

[ref47] Darden T.; York D.; Pedersen L. Particle mesh Ewald: AnN·log(N) method for Ewald sums in large systems. J. Chem. Phys. 1993, 98, 10089–10092. 10.1063/1.464397.

[ref48] Seoane R. G.; Corderí S.; Gómez E.; Calvar N.; González E. J.; Macedo E. A.; Domínguez Á. Temperature Dependence and Structural Influence on the Thermophysical Properties of Eleven Commercial Ionic Liquids. Ind. Eng. Chem. Res. 2012, 51, 2492–2504. 10.1021/ie2029255.

[ref49] Bailey H. E.; Wang Y.-L.; Fayer M. D. Impact of Hydrogen Bonding on the Dynamics and Structure of Protic Ionic Liquid/Water Binary Mixtures. J. Phys. Chem. B 2017, 121, 8564–8576. 10.1021/acs.jpcb.7b06376. 28810731

[ref50] VandeVondele J.; Krack M.; Mohamed F.; Parrinello M.; Chassaing T.; Hutter J. QUICKSTEP: Fast and Accurate Density Functional Calculations Using a Mixed Gaussian and Plane Waves Approach. Comput. Phys. Commun. 2005, 167, 103–128. 10.1016/j.cpc.2004.12.014.

[ref51] Hutter J.; Iannuzzi M.; Schiffmann F.; VandeVodele J. CP2K: Atomistic Simulations of Condensed Matter Systems. WIREs Comput. Mol. Sci. 2014, 4, 15–25. 10.1002/wcms.1159.

[ref52] Grimme S.; Antony J.; Ehrlich S.; Krieg H. A consistent and accurate ab initio parametrization of density functional dispersion correction (DFT-D) for the 94 elements H-Pu. J. Chem. Phys. 2010, 132, 154104 10.1063/1.3382344. 20423165

[ref53] Goedecker S.; Teter M.; Hutter J. Separable Dual-Space Gaussian Pseudopotentials. Phys. Rev. B 1996, 54, 1703–1710. 10.1103/PhysRevB.54.1703. 9986014

[ref54] VandeVondele J.; Hutter J. Gaussian basis sets for accurate calculations on molecular systems in gas and condensed phases. J. Chem. Phys. 2007, 127, 114105 10.1063/1.2770708. 17887826

[ref55] Hourahine B.; Aradi B.; Blum V.; Bonafé F.; Buccheri A.; Camacho C.; Cevallos C.; Deshaye M. Y.; Dumitrică T.; Dominguez A.; Ehlert S.; Elstner M.; van der Heide T.; Hermann J.; Irle S.; Kranz J. J.; Köhler C.; Kowalczyk T.; Kubař T.; Lee I. S.; Lutsker V.; Maurer R. J.; Min S. K.; Mitchell I.; Negre C.; Niehaus T. A.; Niklasson A. M. N.; Page A. J.; Pecchia A.; Penazzi G.; Persson M. P.; Řezáč J.; Sánchez C. G.; Sternberg M.; Stöhr M.; Stuckenberg F.; Tkatchenko A.; Yu V. W. Z.; Frauenheim T. DFTB+, a Software Package for Efficient Approximate Density Functional Theory Based Atomistic Simulations. J. Chem. Phys. 2020, 152, 124101 10.1063/1.5143190. 32241125

[ref56] Gaus M.; Goez A.; Elstner M. Parametrization and Benchmark of DFTB3 for Organic Molecules. J. Chem. Theory Comput. 2013, 9, 338–354. 10.1021/ct300849w. 26589037

[ref57] Kubillus M.; Kubař T.; Gaus M.; Řezáč J.; Elstner M. Parameterization of the DFTB3 Method for Br, Ca, Cl, F, I, K, and Na in Organic and Biological Systems. J. Chem. Theory Comput. 2015, 11, 332–342. 10.1021/ct5009137. 26889515

[ref58] Lukose B.; Kuc A.; Frenzel J.; Heine T. On the reticular construction concept of covalent organic frameworks. Beilstein J. Nanotechnol. 2010, 1, 60–70. 10.3762/bjnano.1.8. 21977395 PMC3045923

[ref59] Vuong V. Q.; Kuriappan J. A.; Kubillus M.; Kranz J. J.; Mast T.; Niehaus T. A.; Irle S.; Elstner M. Parametrization and Benchmark of Long-Range Corrected DFTB2 for Organic Molecules. J. Chem. Theory Comput. 2018, 14, 115–125. 10.1021/acs.jctc.7b00947. 29232515

[ref60] Lourenço M. P.; Dos Santos E. C.; Pettersson L. G. M.; Duarte H. A. Accurate SCC-DFTB Parametrization for Bulk Water. J. Chem. Theory Comput. 2020, 16, 1768–1778. 10.1021/acs.jctc.9b00816. 32040315

[ref61] Grimme S.; Bannwarth C.; Shushkov P. A Robust and Accurate Tight-Binding Quantum Chemical Method for Structures, Vibrational Frequencies, and Noncovalent Interactions of Large Molecular Systems Parametrized for All spd-Block Elements (Z = 1–86). J. Chem. Theory Comput. 2017, 13, 1989–2009. 10.1021/acs.jctc.7b00118. 28418654

[ref62] Bannwarth C.; Ehlert S.; Grimme S. GFN2-xTB- An Accurate and Broadly Parametrized Self-Consistent Tight-Binding Quantum Chemical Method with Multipole Electrostatics and Density-Dependent Dispersion Contributions. J. Chem. Theory Comput. 2019, 15, 1652–1671. 10.1021/acs.jctc.8b01176. 30741547

[ref63] Brehm M.; Thomas M.; Gehrke S.; Kirchner B. TRAVIS – A Free Analyzer for Trajectories from Molecular Simulation. J. Chem. Phys. 2020, 152, 164105 10.1063/5.0005078. 32357781

[ref64] Bertie J. E.; Lan Z. Infrared Intensities of Liquids XX: The Intensity of the OH Stretching Band of Liquid Water Revisited, and the Best Current Values of the Optical Constants of H2O(l) at 25°C between 15,000 and 1 cm^–1^. Appl. Spectrosc. 1996, 50, 1047–1057. 10.1366/0003702963905385.

[ref65] Dhumal N. R.; Noack K.; Kiefer J.; Kim H. J. Molecular Structure and Interactions in the Ionic Liquid 1-Ethyl-3-methylimidazolium Bis(Trifluoromethylsulfonyl)imide. J. Phys. Chem. A 2014, 118, 2547–2557. 10.1021/jp502124y. 24611825

[ref66] Taherivardanjani S.; Elfgen R.; Reckien W.; Suarez E.; Perlt E.; Kirchner B. Benchmarking the Computational Costs and Quality of Vibrational Spectra from Ab Initio Simulations. Adv. Theory Simul. 2022, 5, 2100293 10.1002/adts.202100293.

[ref67] Sun Q. The Raman OH Stretching Bands of Liquid Water. Vib. Spectrosc. 2009, 51, 213–217. 10.1016/j.vibspec.2009.05.002.

[ref68] Liu J.; Lan J.; He X. Toward High-level Machine Learning Potential for Water Based on Quantum Fragmentation and Neural Networks. J. Phys. Chem. A 2022, 126, 3926–3936. 10.1021/acs.jpca.2c00601. 35679610

