# Supplementary material for: Hydrogen Bonding and Infrared Spectra of Ethyl-3-methylimidazolium Bis(trifluoromethylsulfonyl)imide/Water Mixtures: A View from Molecular Dynamics Simulations
Source: J Phys Chem B. 2022 Dec 14;126(51):10922–32. doi: 10.1021/acs.jpcb.2c06947 (PMC9806834; doi:10.1021/acs.jpcb.2c06947)
Supplement: Supplementary file 1 — jp2c06947_si_001.pdf [file jp2c06947_si_001.pdf]

Hydrogen Bonding and IR Spectra of EMIM-TFSI/Water Mixtures – A View from Molecular Dynamics Simulations

Piotr Wróbel, Piotr Kubisiak, Andrzej Eilmes*

Faculty of Chemistry, Jagiellonian University, Gronostajowa 2, 30-387 Kraków, Poland

Supporting Information

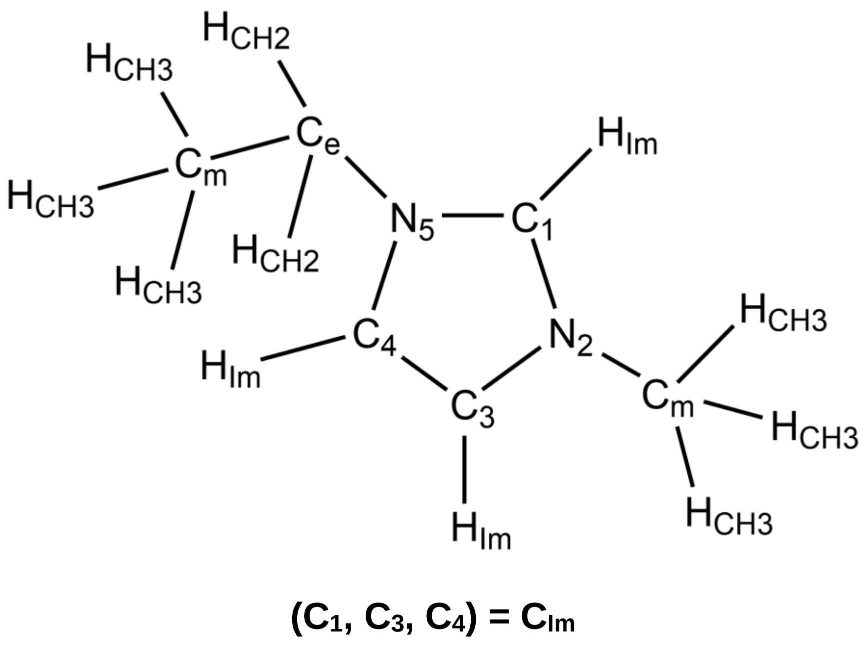

Scheme S1. Labeling of atoms in EMIM cation.

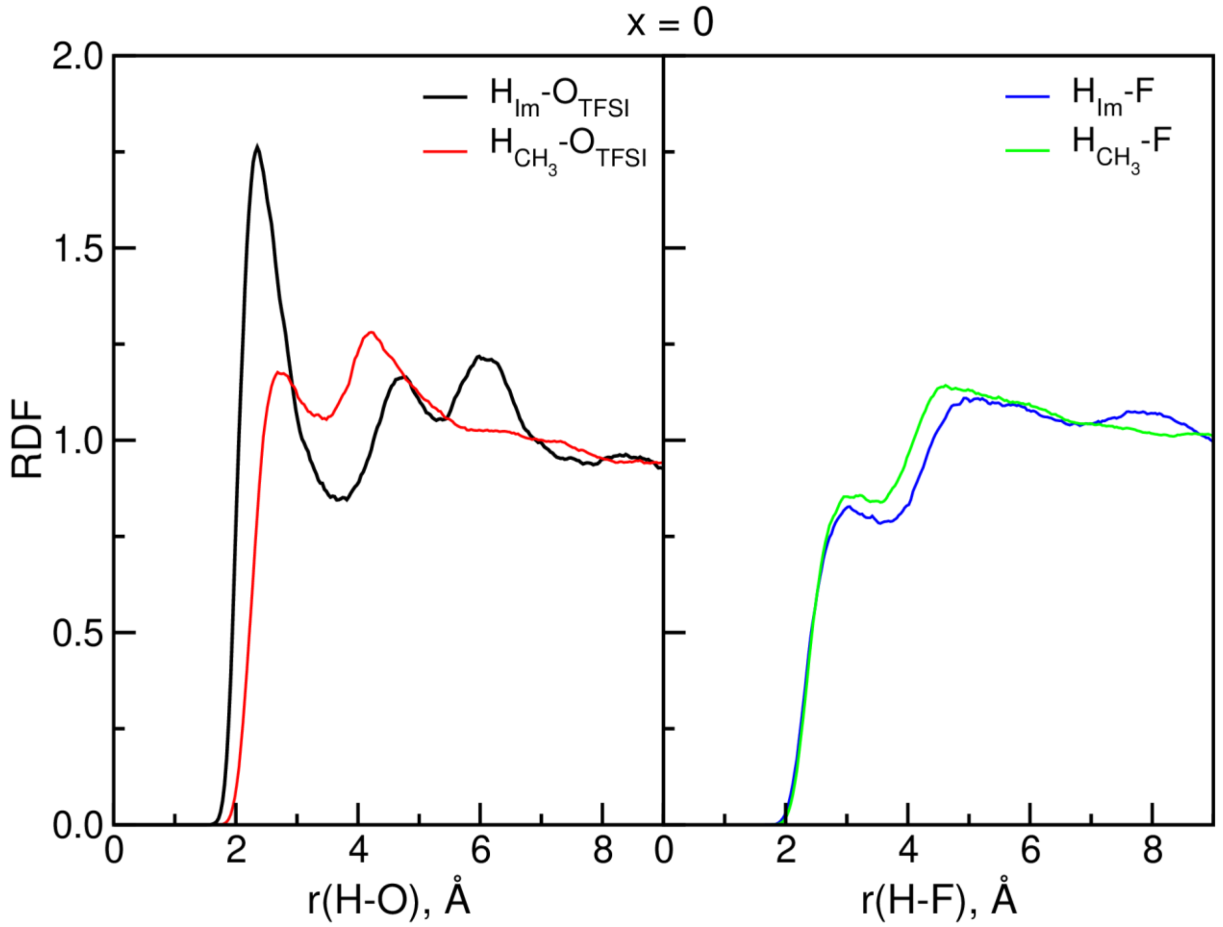

Figure S1. Radial distribution functions for selected atom pairs obtained from AIMD simulations for $x = 0$.

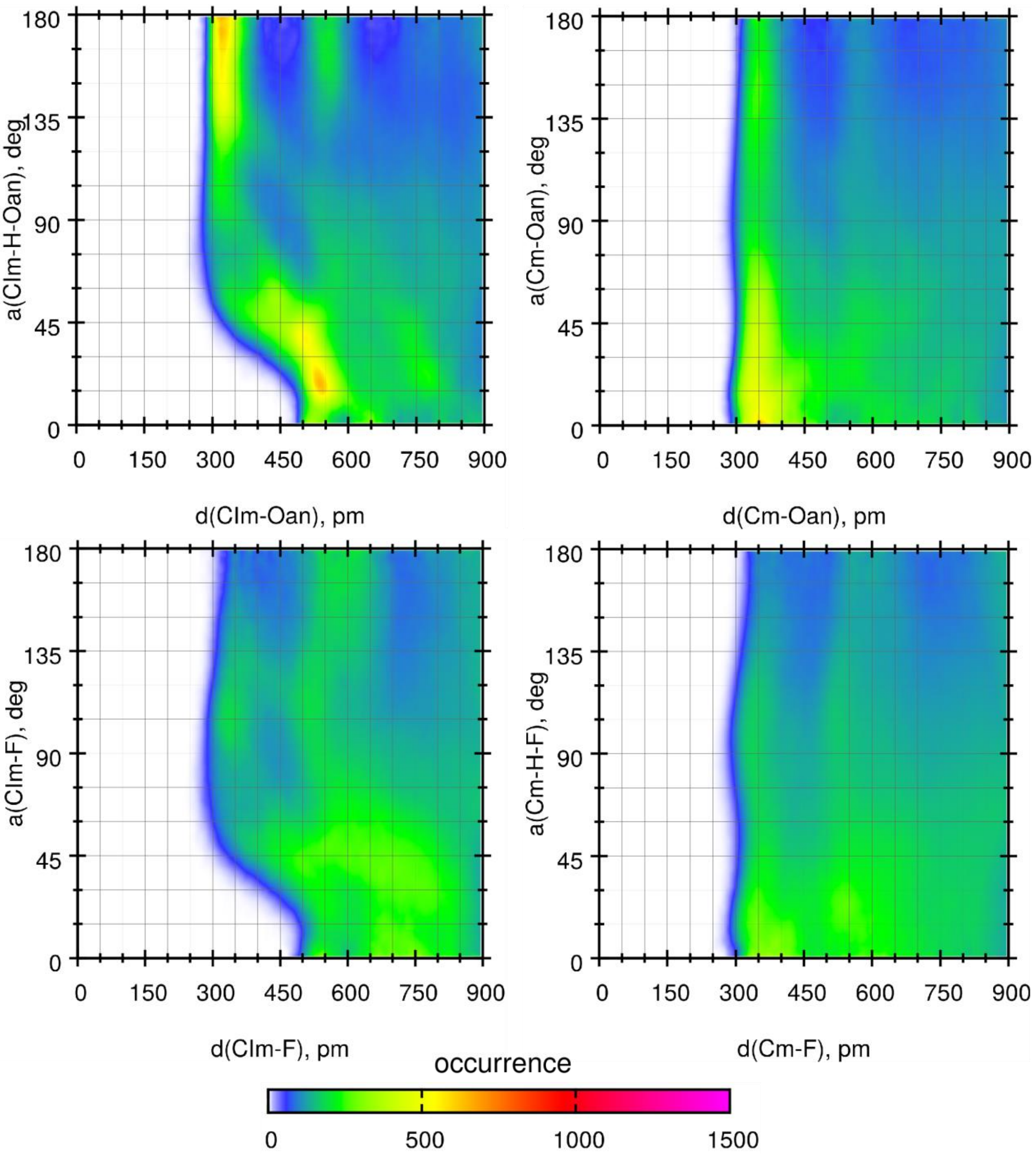

Figure S2. Combined distribution functions for selected D-H-A atoms in the neat IL. Cm denotes C atoms from CH_3 groups, CIm are the C atoms from the imidazolium ring, and Oan are the O atoms from TFSI anions.

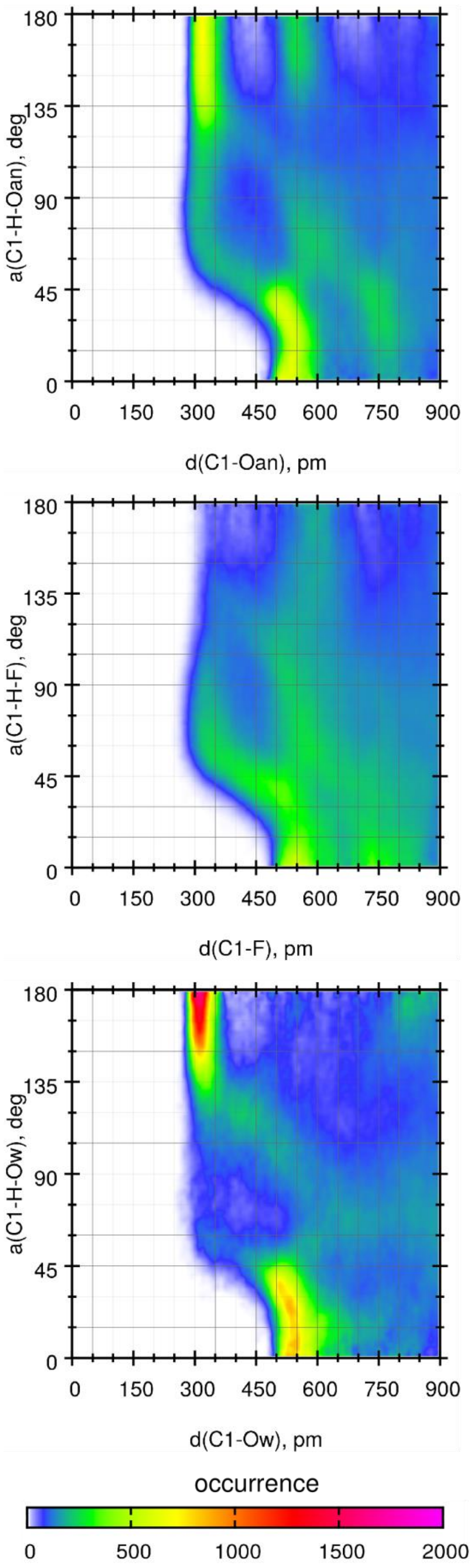

Figure S3. Combined distribution functions involving C_1 atoms in the $x = 0.5$ system.

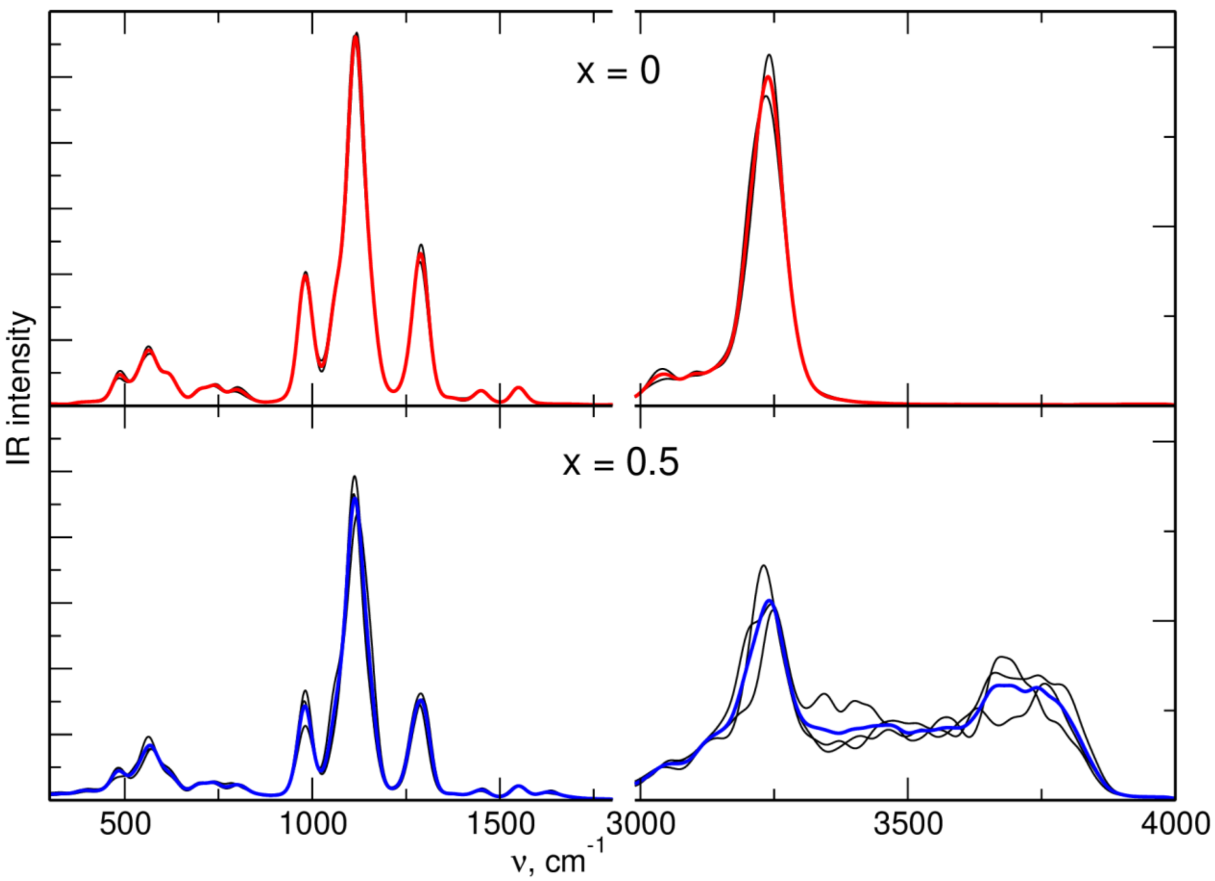

Figure S4. IR spectra calculated for individual replicas of IL/water systems (black lines) and their averages (colored lines).

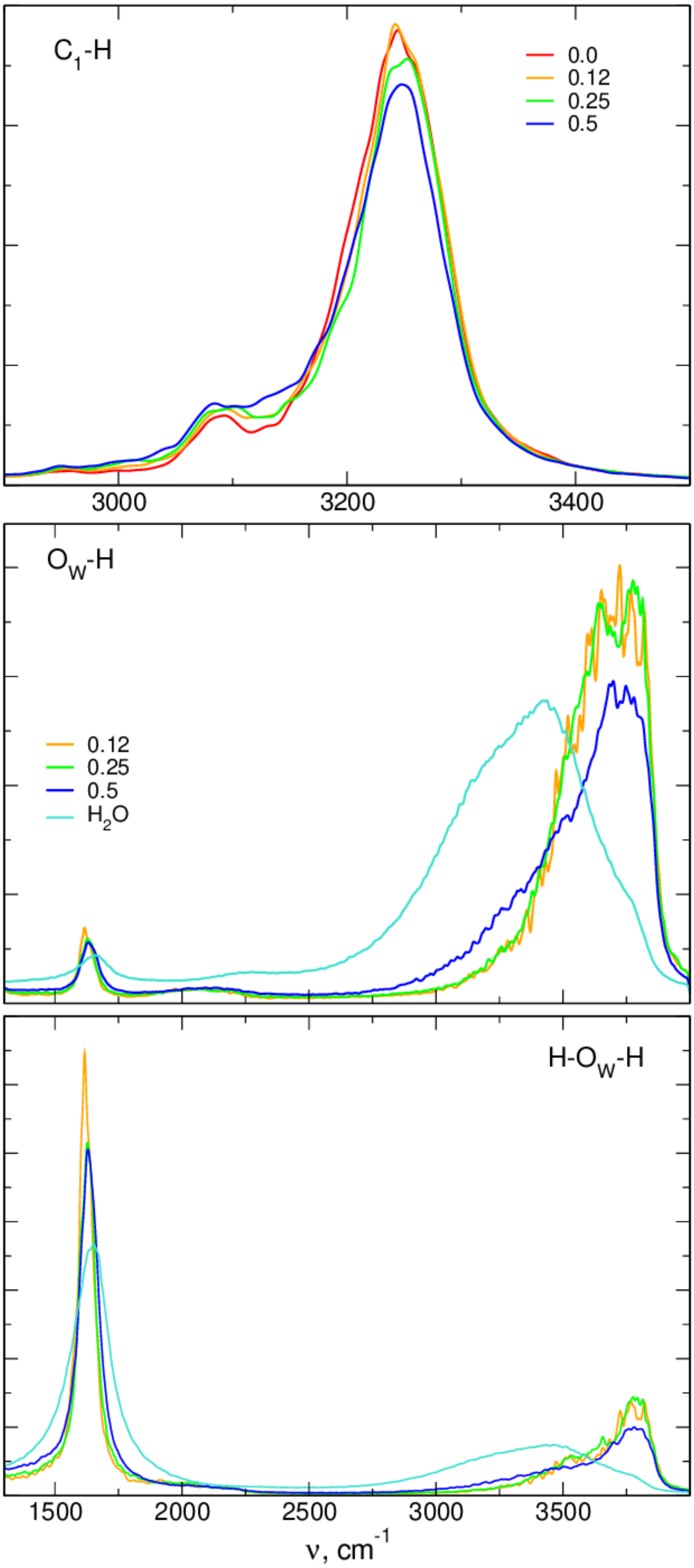

Figure S5. Fourier transforms of C_1-H, O_w-H bond lengths and H-O_w-H angles averaged over all ions/molecules in IL/water mixtures.

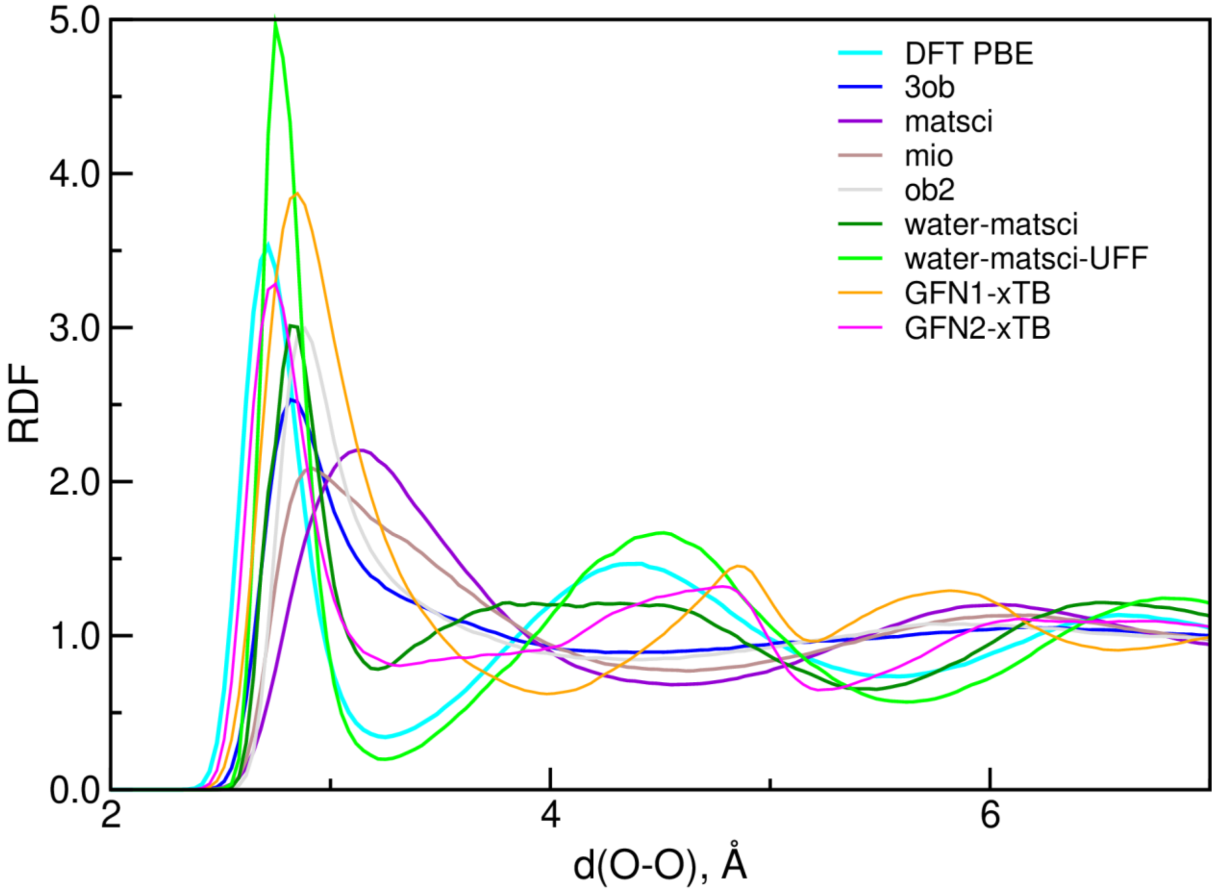

Figure S6. Radial distribution functions for O_w-O_w pairs in bulk water simulated using different parameterizations.

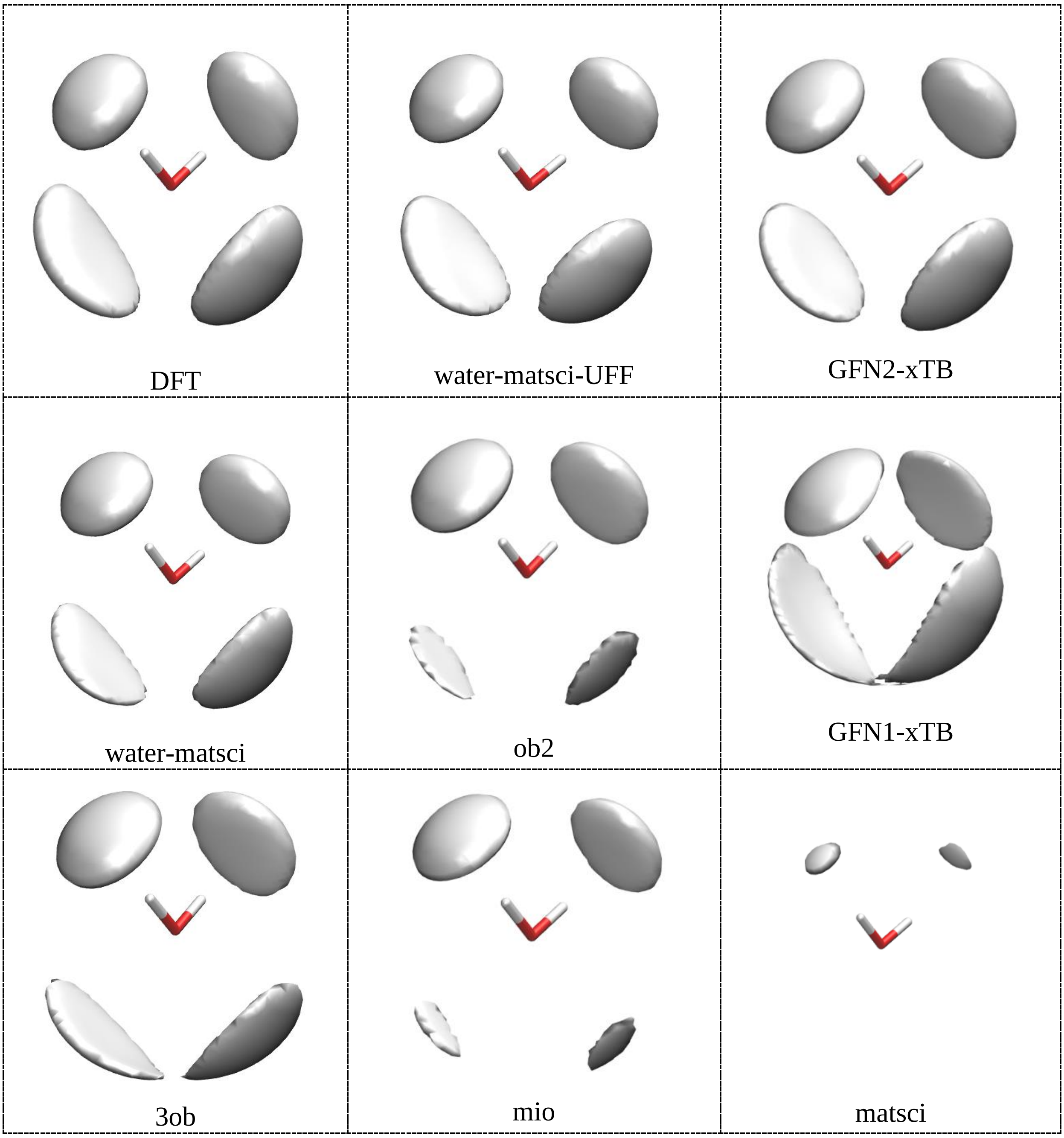

Figure S7. Spatial distribution functions of oxygen atoms around water molecules in bulk water obtained in DFTB simulations. Surfaces of particle density 100 atoms/nm^3 are shown.

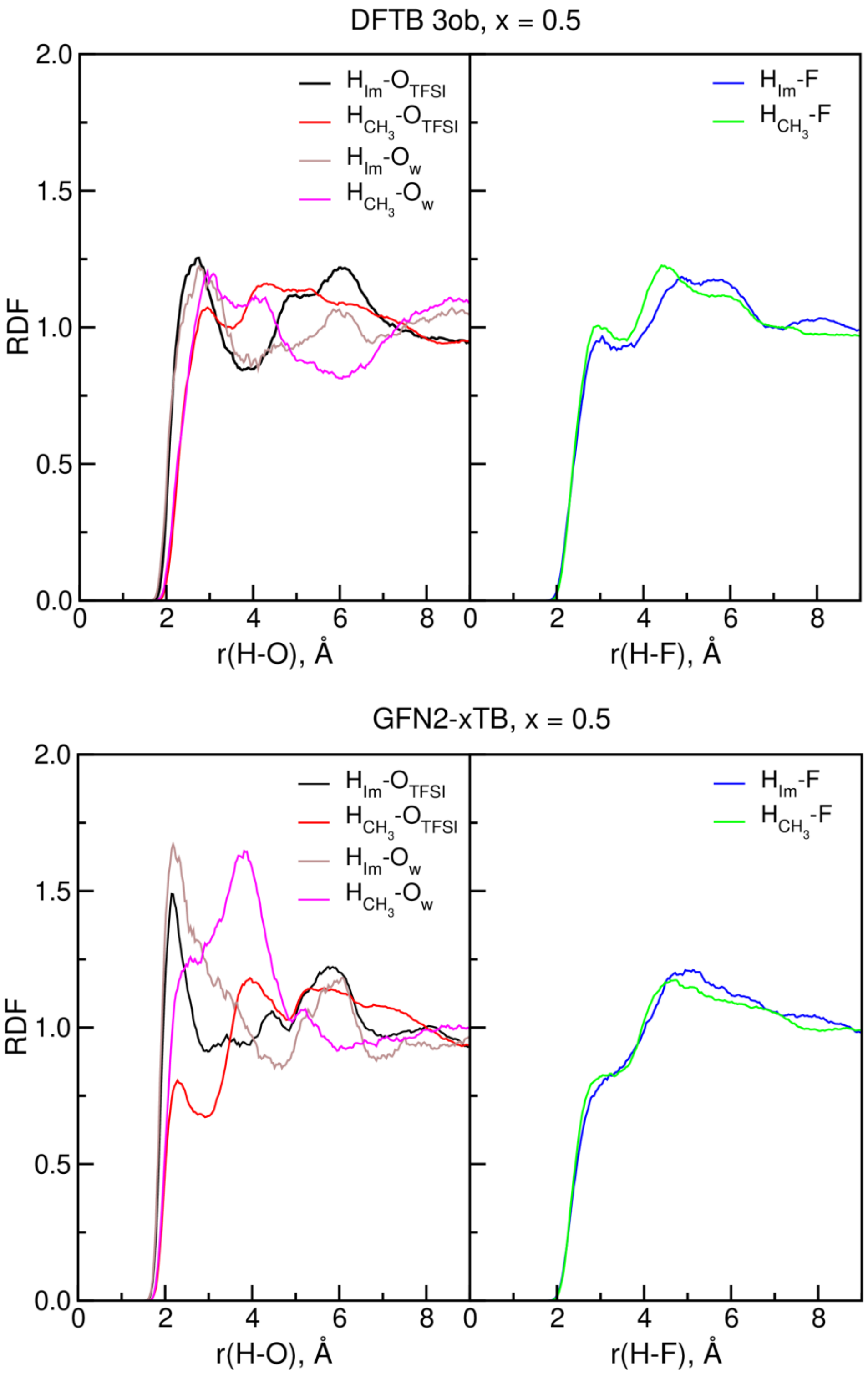

Figure S8. Radial distribution functions for selected atom pairs obtained from DFTB simulations for *x* = 0.5.

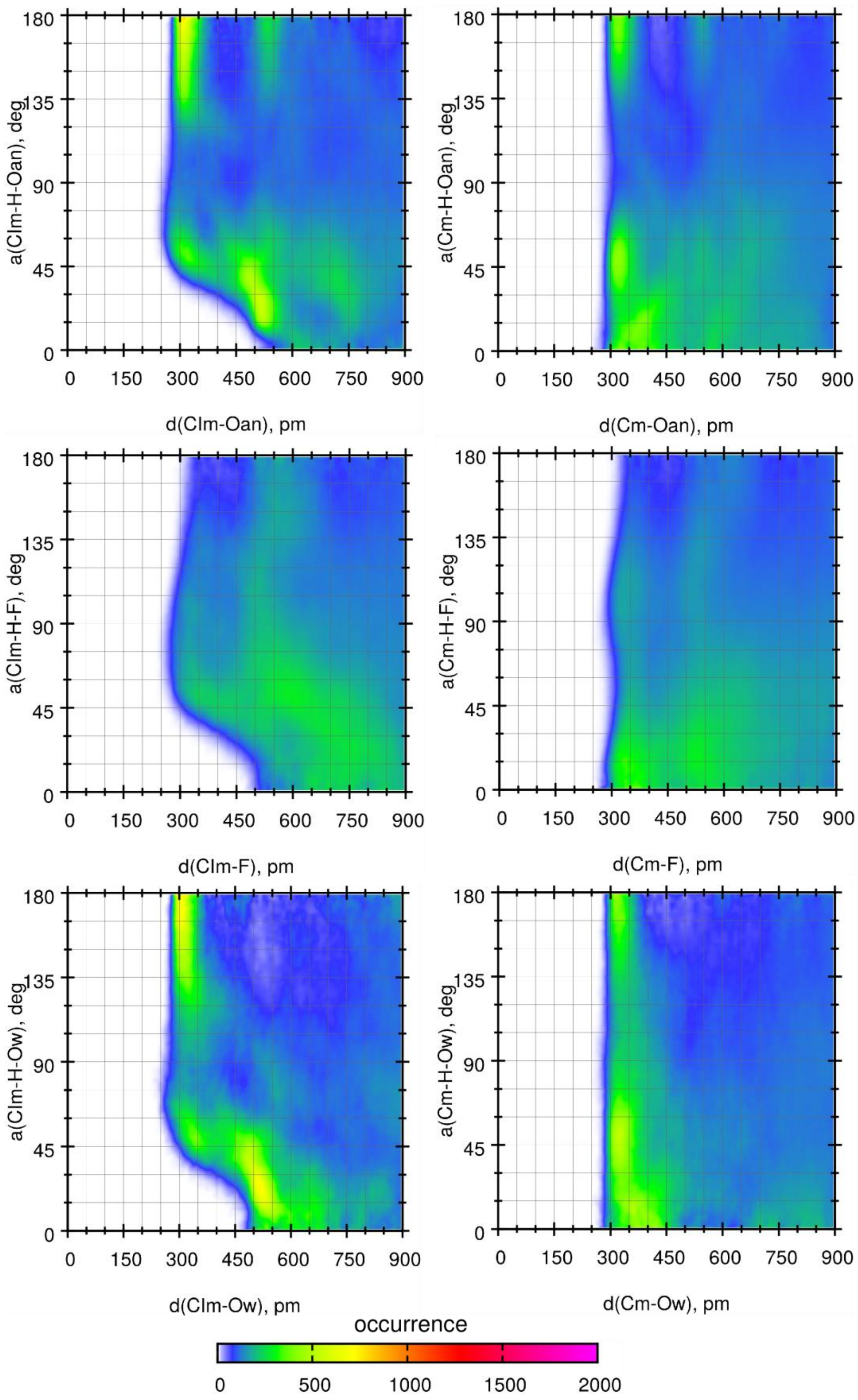

Figure S9. Combined distribution functions for selected D-H-A atoms in the $x = 0.5$ system simulated in GFN2-xTB. Cm denotes C atoms from CH_3 groups, CIm are the C atoms from the imidazolium ring, and Oan are the O atoms from TFSI anions.

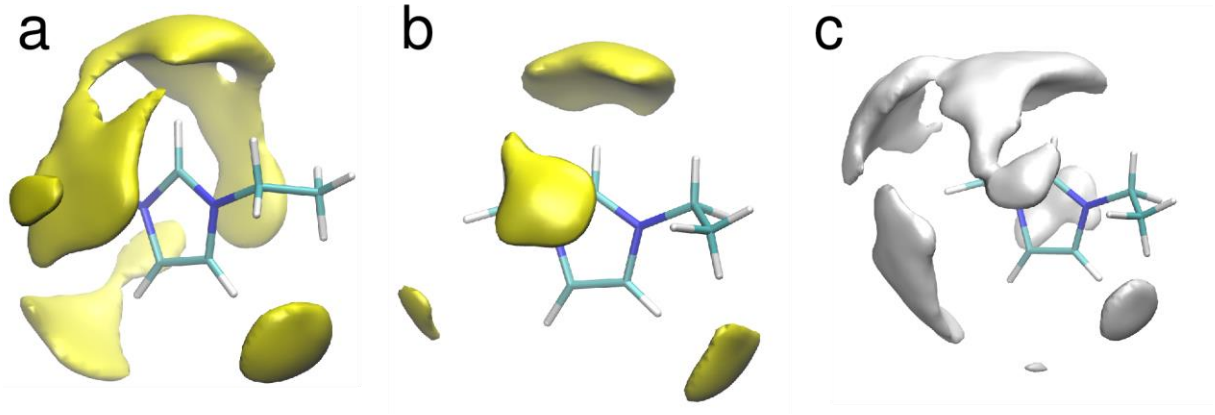

Figure S10. Spatial distribution functions of oxygen atoms around EMIM cations simulated using GFN2-xTB method: TFSI ions in the neat IL (a); TFSI ions in the x = 0.5 mixture (b); water molecules in the x = 0.5 mixture (c). Surfaces of particle density 10 atoms/nm^3 are shown.

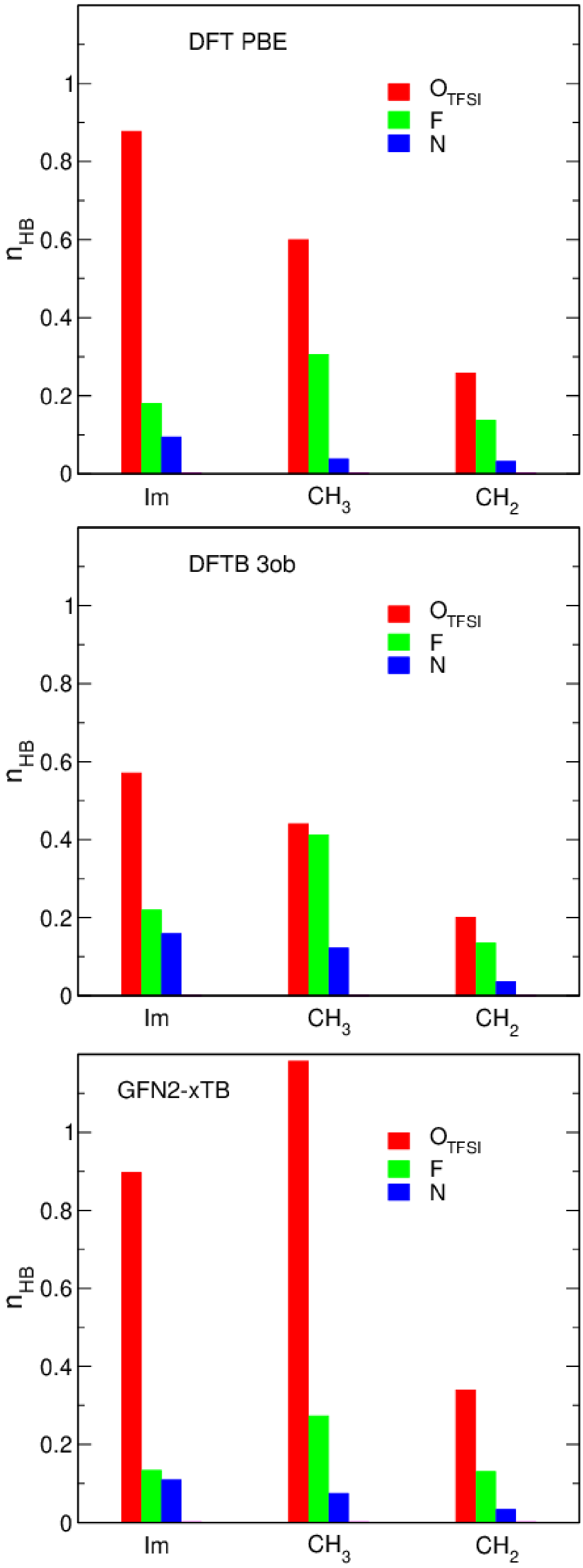

Figure S11. Statistics of hydrogen bonds obtained for x = 0 from MD simulations based on DFT or DFTB. Donors are shown in the horizontal axis; colors mark the acceptors.

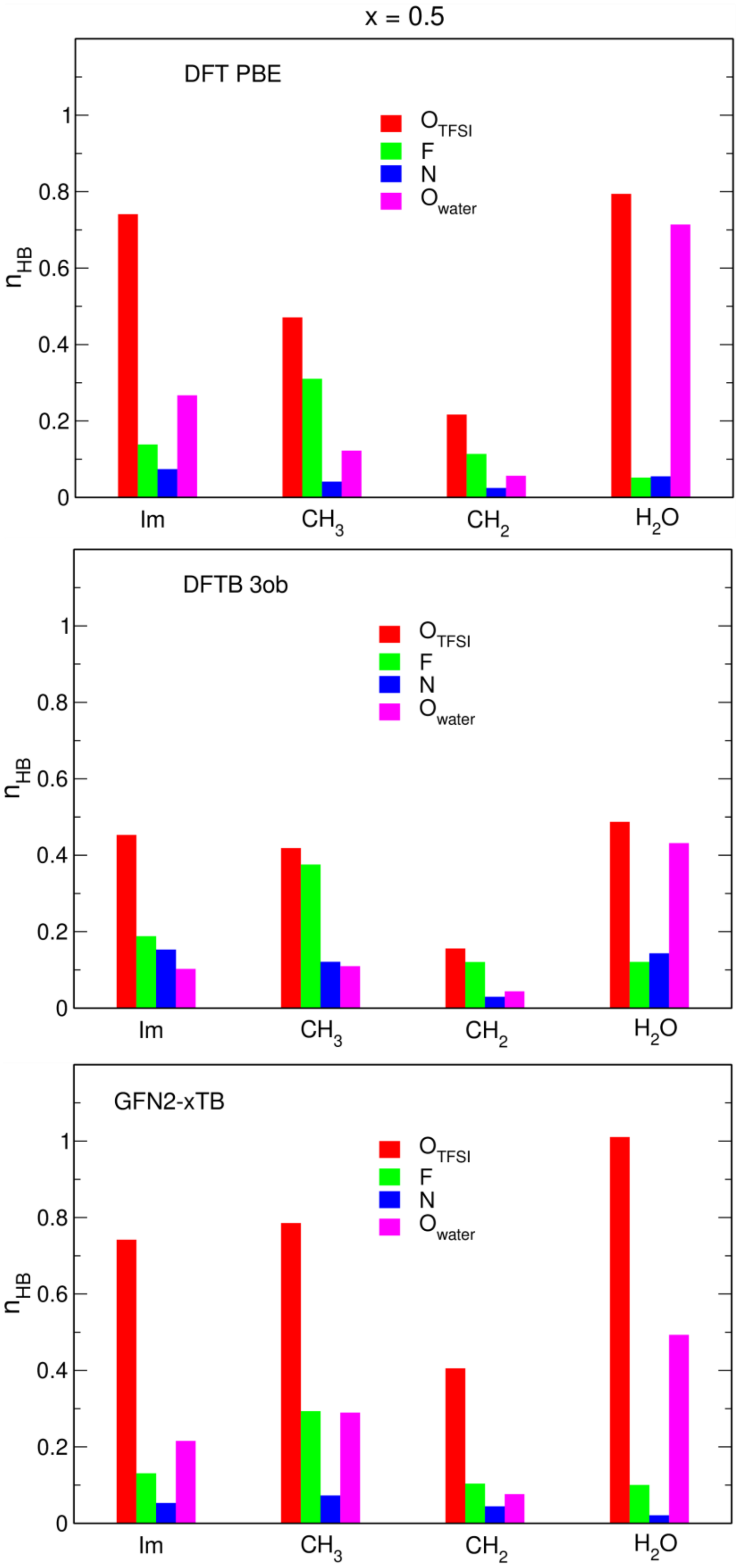

Figure S12. Statistics of hydrogen bonds obtained for $x = 0.5$ from MD simulations based on DFT or DFTB. Donors are shown in the horizontal axis; colors mark the acceptors.

Appendix A. Sample input files.

Sample CP2K input for EMIM-TFSI system

```
&GLOBAL
  PROJECT IL
  RUN_TYPE MD
  PRINT_LEVEL LOW
&END GLOBAL
&FORCE_EVAL
  METHOD Quickstep
  &DFT
    charge 0
    &MGRID
      CUTOFF 260
      NGRIDS 4
      REL_CUTOFF 40
    &END MGRID
    &QS
      EPS_DEFAULT 1.0E-12
      EPS_GVG 1.0E-6
      EPS_PGF_ORB 1.0E-6
    &END QS
    &SCF
      EPS_SCF 1.0E-6
      MAX_SCF 1500
      SCF_GUESS atomic
      &OT
      &END OT
    &END SCF
    &XC
      &XC_FUNCTIONAL PBE
      &END XC_FUNCTIONAL
      &VDW_POTENTIAL
        POTENTIAL_TYPE PAIR_POTENTIAL
        &PAIR_POTENTIAL
          TYPE DFTD3
          REFERENCE_FUNCTIONAL PBE
          PARAMETER_FILE_NAME dftd3.dat
          CALCULATE_C9_TERM T
        &END PAIR_POTENTIAL
      &END VDW_POTENTIAL
    &END XC
    &PRINT
      &MOMENTS SILENT
      &END MOMENTS
    &END PRINT
  &END DFT
  &SUBSYS
    &CELL
      ABC 18.585 18.585 18.585
    &END CELL
    &TOPOLOGY
       &CENTER_COORDINATES T
       &END CENTER_COORDINATES
       COORD_FILE_FORMAT XYZ
       COORD_FILE_NAME IL.xyz
    &END TOPOLOGY
```

```
    &KIND S
      BASIS_SET DZVP-MOLOPT-GTH-q6
      POTENTIAL GTH-PBE-q6
    &END KIND

    &KIND O
      BASIS_SET DZVP-MOLOPT-GTH-q6
      POTENTIAL GTH-PBE-q6
    &END KIND

    &KIND C
      BASIS_SET DZVP-MOLOPT-GTH-q4
      POTENTIAL GTH-PBE-q4
    &END KIND

    &KIND N
      BASIS_SET DZVP-MOLOPT-GTH-q5
      POTENTIAL GTH-PBE-q5
    &END KIND

    &KIND H
      BASIS_SET DZVP-MOLOPT-GTH-q1
      POTENTIAL GTH-PBE-q1
    &END KIND

    &KIND F
      BASIS_SET DZVP-MOLOPT-GTH-q7
      POTENTIAL GTH-PBE-q7
    &END KIND

 &END SUBSYS
&END FORCE_EVAL

&MOTION
  &MD
    ENSEMBLE NVT
    STEPS 40000
    TIMESTEP 1.0
    TEMPERATURE 298
    &THERMOSTAT
      &NOSE
        LENGTH 3
        YOSHIDA 3
        TIMECON 100.
        MTS 2
      &END NOSE
    &END
  &END MD
&END MOTION
```

Sample DFTB+ input for EMIM-TFSI system using 3ob parameterization

```
Geometry = GenFormat {
  <<< "IL.gen"
}

Driver = VelocityVerlet {
  MovedAtoms = 1:-1
  Steps = 40000
  TimeStep [femtosecond] = 1.0
  KeepStationary = Yes
  OutputPrefix = "IL"
  MDRestartFrequency = 1
  Thermostat = NoseHoover {
    Temperature [Kelvin] = 298.0
    CouplingStrength [cm^-1] = 3000.0
    ChainLength = 3
    Order = 3
    IntegratorSteps = 2
  }
}

Hamiltonian = DFTB {
  SCC = Yes
  SlaterKosterFiles = Type2FileNames {
    Prefix = "../3ob-3-1/"
    Separator = "-"
    Suffix = ".skf"
  }
  MaxAngularMomentum {
    O = "p"
    H = "s"
    C = "p"
    N = "p"
    F = "p"
    S = "d"
  }
  Filling = Fermi {
    Temperature [Kelvin] = 298.0
  }
  KPointsAndWeights = SupercellFolding {
    1 0 0
    0 1 0
    0 0 1
    0 0 0
  }
  Dispersion = LennardJones {
    Parameters = UFFParameters {}
  }
  ThirdOrderFull = Yes
  HubbardDerivs {
    O = -0.1575
    H = -0.1857
    C = -0.1492
    N = -0.1535
    F = -0.1623
    S = -0.11
  }
  HCorrection = Damping {
    Exponent = 4.00
  }
```

```
  ForceEvaluation = 'dynamics'
}

Options {
  WriteResultsTag = Yes
}

Analysis = {
  CalculateForces = Yes
}

ParserOptions {
  ParserVersion = 5
}

Parallel = {
  UseOmpThreads = Yes
}
```

Sample DFTB+ input for EMIM-TFSI system using GFN2-xTB method

```
Geometry = GenFormat {
  <<< "IL.gen"
}

Driver = VelocityVerlet {
  MovedAtoms = 1:-1
  Steps = 40000
  TimeStep [femtosecond] = 1.0
  KeepStationary = Yes
  OutputPrefix = "IL"
  MDRestartFrequency = 1
  Thermostat = NoseHoover {
    Temperature [Kelvin] = 298.0
    CouplingStrength [cm^-1] = 3000.0
    ChainLength = 3
    Order = 3
    IntegratorSteps = 2
  }
}

Hamiltonian = xTB {
  Method = "GFN2-xTB"
  SccTolerance = 1e-6
  MaxSccIterations = 1000
  Filling = Fermi {
    Temperature [Kelvin] = 298.0
  }
  KPointsAndWeights = SupercellFolding {
    1 0 0
    0 1 0
    0 0 1
    0 0 0
  }
}

Options {
  WriteResultsTag = Yes
  WriteChargesAsText = Yes
  ReadChargesAsText = Yes
}

Analysis = {
  CalculateForces = Yes
}

ParserOptions {
  ParserVersion = 5
}

Parallel = {
  UseOmpThreads = Yes
}
```
